# *Toxoplasma gondii*-induced region-specific disintegration in glutamatergic neurotransmission is linked to cognitive impairments in mice

**DOI:** 10.1016/j.isci.2025.114415

**Published:** 2025-12-18

**Authors:** Iurii Savvateev, Lorena Morton, Henning Peter Düsedau, Johannes Steffen, Sanja Mikulovic, Dirk Montag, Björn H. Schott, Ildiko Rita Dunay

**Affiliations:** 1 Institute of Inflammation and Neurodegeneration, Health Campus Immunology, Infectiology and Inflammation (GC-I^3^), Otto-von-Guericke University, Magdeburg, Germany; 2 Leibniz Institute for Neurobiology, Department of Behavioral Neurology, Magdeburg, Germany; 3 Center for Behavioral Brain Sciences (CBBS), Magdeburg, Germany; 4 Department of Psychiatry and Psychotherapy, University Medical Center Göttingen, Göttingen, Germany; 5 Leibniz Institute for Neurobiology, Neurogenetics Laboratory, Magdeburg, Germany; 6 Center for Intervention and Research on Adaptive and Maladaptive Brain Circuits Underlying Mental Health (C-I-R-C), German Center for Mental Health (DZPG), Jena-Magdeburg-Halle, Germany

**Keywords:** Immunology, Parasitology, Neuroscience, Cognitive neuroscience

## Abstract

Chronic infection with the protozoan parasite *Toxoplasma gondii* (*T*. *gondii*) elicits distinct alterations in both the immune and nervous system of the host. Previous studies correlated the persistent neuroinflammatory response triggered by chronic *T*. *gondii* infection to specific behavioral alterations. Here, we causally link chronic cerebral *T*. *gondii* infection to cognitive and motor impairments in mice, as well as to the altered brain glutamatergic signaling in hippocampus, striatum, and cortex. By combining synaptic composition analysis assessed via flow synaptometry with the standard sulfadiazine treatment, we demonstrated the regional specificity of the detected alterations of cerebral *T*. *gondii* infection. Importantly, our behavioral analysis exposed the restoration of behavioral flexibility in shifting between goal-directed and habitual action control, with more motorically demanding skills such as social novelty recognition and locomotion being only partially restored. We argue that the revealed regional effects of both *T*. *gondii* and sulfadiazine treatment may be a key factor accounting for treatment-resistant behavioral traits.

## Highlights

• *T. gondii* causes neuroinflammation and hampers Glu synaptic neurotransmission
• Synapses are impaired at structural and functional levels with a regional tropism
• *T. gondii* hinders motor actions, social interaction, goal-directed/habitual balance
• Sulfadiazine causes only a partial restoration of neurotransmission and behavior

## Graphical abstract

**Figure:**
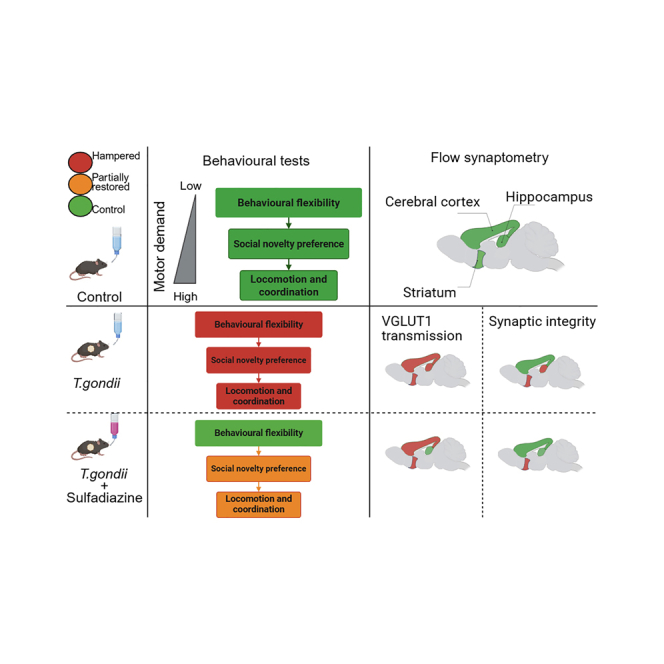

## Introduction

Toxoplasmosis is caused by an intracellular parasite *Toxoplasma gondii* (*T*. *gondii*) considered to be one of the most prevalent infections worldwide.^1^^,^^2^ *T*. *gondii* is able to infect all warm-blooded organisms while directly crossing vital biological barriers within the body, including the intestinal, blood-brain, and placental barriers.^3^^,^^4^^,^^5^^,^^6^ During latent toxoplasmosis, the immune response induces the transformation of *T*. *gondii* from its rapidly replicating tachyzoite form to slowly dividing bradyzoites within tissue cysts.^7^ Upon infiltrating the central nervous system (CNS), the parasites remain inside neurons lifelong, with no existing medication capable of entirely eliminating this dormant stage.^8^ The conventional antimicrobial agent sulfadiazine, in combination with other anti-parasitic drugs, remains the gold standard for treating the active stage of this disease.^9^

Latent toxoplasmosis in humans has been associated with a spectrum of behavioral alterations, including altered goal-directed behavior^10^^,^^11^ and an increased risk of severe neuropsychiatric conditions, such as epilepsy,^12^^,^^13^ schizophrenia,^12^^,^^14^^,^^15^^,^^16^ bipolar disorder,^15^^,^^16^ and obsessive-compulsive disorder (OCD).^17^ It must be cautioned, though, that studies linking abnormalities in humans to *T*. *gondii* infection largely rely on correlations rather than establishing causality.^18^ Animal models, particularly various murine models of *T*. *gondii* infection, can help to provide insight into the underlying immunological and neural processes.^1^^,^^19^^,^^20^^,^^21^^,^^22^^,^^23^^,^^24^^,^^25^^,^^26^ Infection with *T*. *gondii* causes a rapid immune response to protect the host against the pathogen; however, it also triggers an imbalance in immune homeostasis and dysregulation of neural function.^8^^,^^27^ In the context of latent toxoplasmosis, the host’s immune system must continuously suppress the potential reactivation of bradyzoites, thus promoting an ongoing basal neuroinflammation.^1^^,^^7^^,^^20^^,^^28^

Our previous investigations in murine toxoplasmosis models revealed that *T*. *gondii-*induced neuroinflammation results in structural changes within neurons, as evidenced by reduced neuronal connectivity, decreased dendritic length and complexity and synaptic deficits,^29^ manifested by downregulation of key pre/postsynaptic markers such as synaptophysin and PSD95,^29^ as well as alterations in AMPA and NMDA receptors.^30^ These findings are in line with other papers describing changes in glutamatergic^31^^,^^32^^,^^33^ and GABAergic^34^^,^^35^ neurotransmission. In the present study, we linked *T*. *gondii* ME-49-strain-induced neuroinflammation to changes in synaptic neurotransmission and broad behavioral alterations in C57BL/6J mice. In addition, considering the existing controversy regarding whether definite behavioral alterations upon *T*. *gondii* infection depend on ongoing neuroinflammation,^21^^,^^24^ we used an anti-parasitic drug, sulfadiazine, to indirectly mitigate neuroinflammation. Sulfadiazine treatment facilitates the regulation of the immune response in the CNS but only partially reverses the observed changes in excitatory synaptic transmission.

## Results

### Behavioral alterations caused by *T*. *gondii* infection

We used a combination of behavioral tests that allowed us to discern higher-order cognitive dysfunction and impaired motor capabilities. Specifically, we employed open field, induced grooming, and marble burying tests to characterize the innate motor behavior (explorative locomotion, digging, and grooming, respectively), whereas social interaction (SI) test and a custom-developed behavioral flexibility test were used to assess the effects of *T*. *gondii* infection on cognition such as alterations in social novelty preference and the ability to flexibly shift between goal-directed and habitual action control.

### *T*. *gondii* provokes a lack of social novelty preference

**Figure 1:**
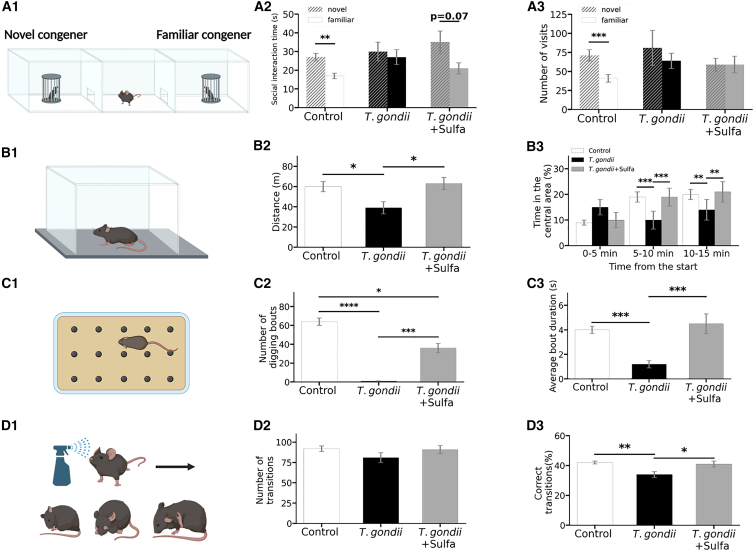
Complex effects of *T*. *gondii* infection and subsequent sulfadiazine treatment on locomotion, motor coordination, and social interactions Schematic illustration of the behavioral paradigms. (A1) Social interaction. (B1) Open field. (C1) Marble burying. (D1) Induced grooming. Principal results of the behavioral tests. (A2 and A3) Social interaction test. (A2) Time spent for the social interaction. Social interaction duration of the experimental mouse with either familiar (solid bars) or novel (dashed bars) congener is depicted. Bars represent the time spent during the social contact (snuffing of the stranger by the experimental mouse). (A3) Number of visits to the social interaction zone. Number of visits into the social interaction zone of either familiar (solid bars) or novel (dashed bars) congener made by the experimental mouse is shown. (B2 and B3) Open field (OF) test. (B2) Total distance traveled during OF. Bars represent the average total distance traveled by each experimental group. (B3) Exploration strategy during OF. Time spent in the central area of the open field (%) during three consecutive 5-min intervals is shown. (C2 and C3) Marble burying test. Plots depict the parameters of the digging activity measured for each experimental group at marble burying test (C1): number of digging bouts (C2) and average bout duration (C3). (D2–D4) Induced grooming test. Parameters of the grooming activity measured for each experimental group during the induced grooming test (D1): number of transitions (D2), percentage of correct transitions (D3). Bars represent mean values ±standard error of the mean (∗∗∗∗*p* < 0.0001 ∗∗∗*p* < 0.001 ∗∗*p* < 0.01 ∗*p* < 0.05). The exact statistical values along with the used tests are shown in Tables S1, S2, S4, S6, and S7. Figures 1A1, B1, C1, and D1 were created with BioRender.

As social animals, mice naturally display a tendency to interact with their congeners. With SI test, we analyzed the preference of a mouse to spend time with either a novel congener or a familiar one^36^ (Figure 1A1; Figure S1; STAR Methods).

Here, we observed that the uninfected control animals (control) spent more time with the novel congener than the familiar congener (t(25) = 3.46, *p* = 0.002), whereas *T*. *gondii*-infected mice demonstrated no preference (t(21) = 0.38, *p* = 0.7) (Figure 1A2), thereby exposing a lack of preference to social novelty. Sulfadiazine-treated mice (*T*. *gondii*+Sulfa) displayed a trend toward spending more time with the novel compared to the familiar congener (t(16) = 1.97; *p* = 0.07) resembling the control group performance (Figure 1A2). However, the *T*. *gondii*+Sulfa group made the same number of visits to the novel and familiar congeners (t(16) = -0.04, *p* = 0.97) similar to the *T*. *gondii* group (Z = −0.11, *p* = 0.9) but in contrast to the control group, which exhibited a preference for visiting novel congeners more frequently (t(25) = 3.1, *p* = 0.005) (Figure 1A3,full statistics, see Table S1). As the number of visits to the novel congener reflects exploratory locomotion, we concluded that sulfadiazine treatment of *T*. *gondii*-infected mice did not restore the exploration strategy. During the social interaction assessment, there were no statistically significant differences in distance traveled between the control, *T*. *gondii*, and *T*. *gondii*+Sulfa groups (Figure S1, χ2(2) = 4.48, *p* = 0.11). This suggests that potential physical debilities were likely not the main factors driving differences observed in this behavioral paradigm.

Taken together, *T*. *gondii* infection impaired social novelty interactions in mice, and anti-parasitic treatment with sulfadiazine could not fully restore this impairment. Further, the social novelty exploration strategy was impaired in both the *T*. *gondii* and *T*. *gondii*+Sulfa groups. Importantly, the abovementioned social interaction impairments described are not directly linked to prospective motor disabilities.

### Chronic *T*. *gondii* infection hinders locomotion and affects exploration

The analysis of overall locomotor activity (Figure 1B1) demonstrated that *T*. *gondii*-infected animals traveled less distance (39 m ± 6 m) compared to the control (60 m ± 5 m) and *T*. *gondii*+Sulfa (63 m ± 6 m) groups (Figure 1B2; Table S2).

The time spent in the central area of the open field during each of the 5-min intervals (0–5 min, 5–10 min, 10–15 min) was determined to examine the exploration strategy. The typical exploration pattern of C57BL/6 mice during the establishment of a “home base”^37^ shows an increase in the time spent in the central area with progressing test duration.^38^ Both control and *T*. *gondii*+Sulfa groups spent more time in the center of the arena during the last 5-min interval (10–15 min) in comparison to the first (0–5 min), whereas the *T*. *gondii* group showed no changes (Figure S2; Table S3). Control and *T*. *gondii*+Sulfa groups displayed a similar preference for the central area during each of the 5-min intervals, suggesting the same exploration strategy. In contrast, the *T*. *gondii* group differed significantly from both control and *T*. *gondii*+Sulfa groups during 5–10 and 10–15 intervals, pointing at a different exploration strategy used by the mice from the *T*. *gondii* group (Figure 1B3; Table S4). Importantly, since the *T*. *gondii* group did not spent more time in the corner areas of the open field (Table S5), we did not attribute this difference in the exploration strategy to elevated anxiety.^38^ Instead, animals explored the novel surroundings less extensively due to a general decrease in locomotion, which is supported by less distance traveled by *T*. *gondii* group compared to control and *T*. *gondii* +Sulfa groups (Table S2).

In line with this observation, the OF test results further demonstrated the amelioration of *T*. *gondii*-induced locomotion debilities by sulfadiazine treatment.

### Chronic *T*. *gondii* infection impairs stereotyped digging behavior

Marble burying assessment (Figure 1C1) exploits the natural tendency of mice to dig in their environment and was performed as described previously^39^^,^^40^^,^^41^ to analyze stereotypic digging behavior and locomotion.

Animals within the *T*. *gondii* group exhibited fewer (Figure 1C2) and shorter (Figure 1C3) digging bouts in comparison to control and *T*. *gondii*+Sulfa groups. Also, the *T*. *gondii* group initiated digging activity later (Figure S3A) and spent less time digging (Figure S3B) compared to control and *T*. *gondii*+Sulfa groups. Together with OF results, these data further support the suppression of locomotion and stereotypical behavior in the *T*. *gondii* group (Table S6). Although control and *T*. *gondii*+Sulfa groups showed the same average bout duration, latency to onset, and time spent digging (Figure 1C3; Figures S3A and S3B), non-infected animals performed more digging bouts (64 ± 4) compared to *T*. *gondii*+Sulfa (36 ± 5) (Q = 3.7, *p* = 0.03) (Figure 1C2), suggesting the remaining activity suppression.

Our results from the marble burying test indicate the suppression of innate stereotypic digging behavior and locomotion upon *T*. *gondii* infection. Sulfadiazine treatment restored the stereotyped digging pattern to the level of non-infected controls, whereas the improvements in locomotion were still below the non-infected level.

### *T*. *gondii* infection impairs cephalocaudal adherence of grooming preserving overall activity and complexity

In the current study, we induced artificial grooming by misting animals with pure water at room temperature (approximately 21°C) (Figure 1D1) and further analyzed the cumulative grooming microstructure as previously described by Kalueff et al.^42^

The number of grooming bouts did not differ among the experimental groups, suggesting an intact ability to initiate grooming activity (F(2,60) = 1.2, *p* = 0.3) (Figure 1D2). Equal numbers of transitions between the different grooming phases displayed by the three groups indicated a similar complexity of the resulting grooming patterns (F_(2,60)_ = 1.7, *p* = 0.19) (Table S7). Grooming patterns of the *T*. *gondii* group demonstrated less adherence to the cephalocaudal direction, as revealed by the lower percentage of correct transitions between the grooming phases (34% ± 2%) compared to control (42% ± 1%) and *T*. *gondii*+Sulfa (41% ± 2%) (F_(2,60)_ = 6.9, *p* = 0.002) (Figure 1D3; Table S7). In addition, mice of the *T*. *gondii* group spent more time grooming (220 ± 20 s) compared to the animals from control (170 ± 10 s) and *T*. *gondii*+Sulfa groups (150 ± 15 s) (F_(2,60)=_5.05, *p* = 0.009) (Figure S4; Table S7). The increase in grooming time accompanied by the unchanged number of grooming bouts implies a prolonged duration of a single grooming bout. Longer bouts with the suppressed adherence to the cephalocaudal direction can be regarded as indicative of an increased anxiety and/or general problems in complex motor coordination.^43^ However, increased anxiety would have led to a regional tropism (e.g., more time in the corners compared to the center) in the open field test.^38^ This was not observed in our results (Table S5).

In summary, our results of the induced grooming test suggest *T*. *gondii* induced alterations in the complex motor coordination, which can be restored by sulfadiazine treatment.

### *T*. *gondii* infection impaired task-dependent shift between goal-directed and habitual action controls

While the results of the behavioral tests described earlier allowed us to pinpoint behavioral alterations in motor and social interaction behaviors, we next sought to further investigate the effects of *T*. *gondii* infection on the fundamental ability to flexibly shift between goal-directed and habitual behaviors. Such flexibility is pivotal, and its impairments are linked to various neurological conditions including addiction and OCD.^44^^,^^45^^,^^46^ In the following experiments, we investigated whether *T*. *gondii* alters behavioral flexibility and, therefore, supports a causative connection between *T*. *gondii* and one of the key disturbances behind the decision-making pathologies.

To test behavioral flexibility, we adapted a previously developed instrumental nose-poking paradigm by allowing a context-dependent segregation between goal-directed and habitual action controls.^47^ In short, reinforcement schedules that favor either goal-directed or habitual behavior, random ratio (RR), or random interval (RI), respectively, are paired with a certain contextual cue. Mice are water-deprived and trained to nose-poke under RR and RI (on the same day) to get water. Therefore, the execution of goal-directed versus habitual behavior is coupled with distinct contexts. The training schedule facilitates the further segregation of the behavior, by increasing ratio and interval values. If an animal’s flexibility to shift between these two behavioral modes is intact, one can observe segregation between goal-directed and habitual modes, depending on the context, at the end of the experiment. Finally, to probe the extent to which actions are controlled through goal-directed or habitual behavior, the outcome devaluation procedure (mice have *ad libitum* access to water, and nose pokes are not rewarded in the reinforcement delivery) is performed, and non-rewarded pokes are measured (Figure S5; Table S8). The goal-directed behavior is linked to a response-outcome association and, therefore, decreased once the reinforcement is devaluated or removed (i.e., outcome devaluation), whereas the habitual regime is governed by stimulus-response associations lacking the link to the outcome and should not be affected by the devaluation.^44^

**Figure 2:**
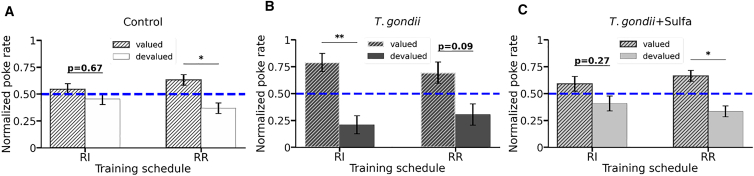
Behavioral flexibility test The normalized poke rates for (A) control, (B) *T*. *gondii*, and (C) *T*. *gondii*+Sulfa groups in two different contexts: Random interval (RI) and random ratio (RR) for valued and devalued days. Bars represent the mean values ± standard error of the mean. The dashed line at 0.5 depicts the level at which poke rates for valued and devalued days are equal; the further the spacing from 0.5, the higher the difference between the performances. In cases where the differences between the poke rates on valued and devalued days were not statistically significant, the exact *p* values are shown.∗*p* < 0.05; ∗∗*p* < 0.01. The exact statistical values along with the used tests are shown in Table S9.

During the devaluation test, both control and *T*. *gondii*+Sulfa groups showed a decrease in the poke rate only in the context previously coupled with the goal-directed training schedule, but not in the context used during the habitual reinforcement, indicating that behavioral flexibility was preserved (Figures 2A and 2C). Meanwhile, animals in the *T*. *gondii* group did not show a statistically significant decrease in a normalized poke rate within the goal-directed context. Instead, they exhibited a reduction in the context previously associated with the habitual schedule (Figure 2B), indicating an altered transition between goal-directed and habitual action control (see Table S9 for full statistics). At the same time, from the analysis of the performance intensity (Figures S6A1–A3) and general activity (Figures S6B1–B3) between the RR and RI reinforcement schedules, we concluded, similarly as done earlier for a lever-press paradigm,^47^^,^^48^ that both schedules promote the same physical patterns of the poking behavior. However, as detailed above, these patterns are generated by applying different behavioral strategies (Figure S7).

Thus, the *T*. *gondii* group could not flexibly shift between goal-directed and habitual schedules, whereas control and *T*. *gondii*+Sulfa groups flexibly adapted their behavior.

### Alteration of excitatory neurotransmission by *T*. *gondii*

**Figure 3:**
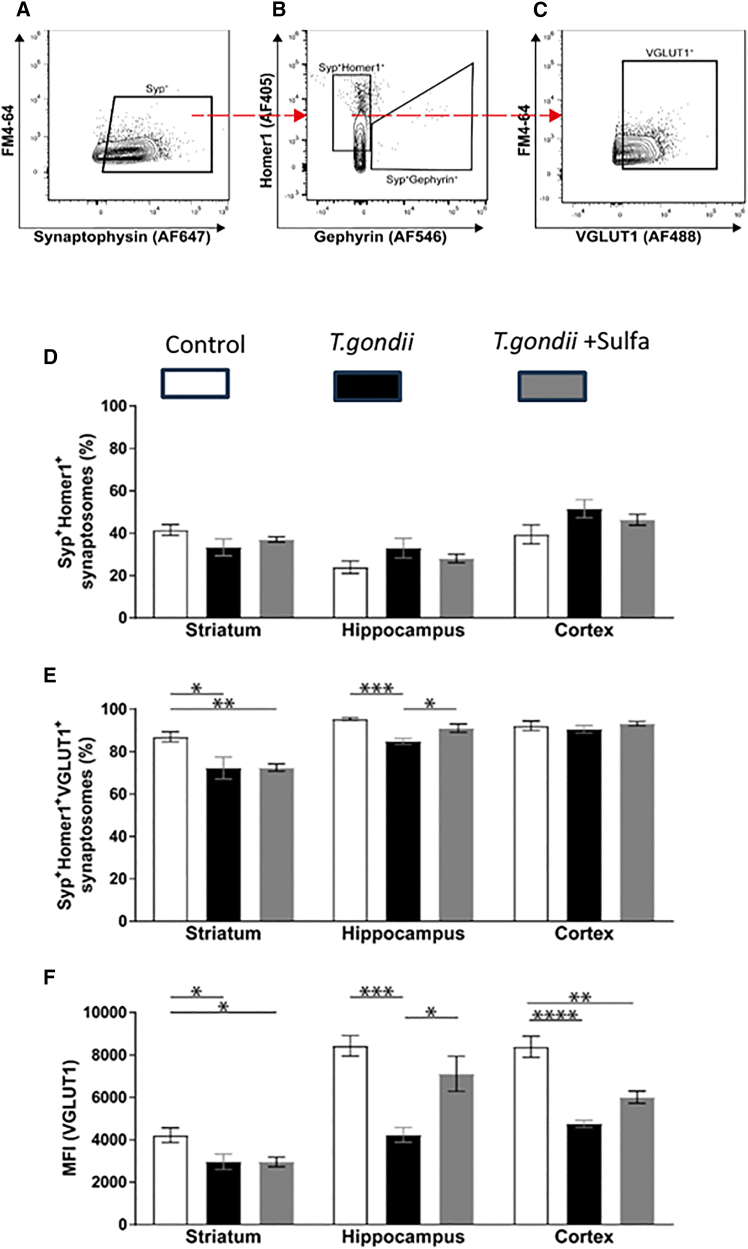
Region-specific flow synaptometry (A–C) Representative gating strategy for flow synaptometric analysis of isolated synaptosomes is shown. (A) Synaptophysin was used to select synaptosomes. (B) Postsynaptic proteins Homer1 and Gephyrin were used to separate synaptosomes formed by excitatory versus inhibitory synapses. (C) Vesicular glutamate transporter protein 1 (VGLUT1) served as a presynaptic marker to verify the selection of synaptosomes formed by excitatory synapses. (D) Percentage of the extracts positive for Homer1 and Synaptophysin from the parent populations. (E) Percentage of the extracts positive for Homer1, Synaptophysin, and VGLUT1. (F) Median fluorescent intensity (MFI) of VGLUT1-positive synaptosomes from extracts of synaptosomes positive for Homer1, Synaptophysin, and VGLUT1. Bars represent the mean values ± standard error of the mean. ∗∗∗∗*p* < 0.0001, ∗∗∗*p* < 0.001, ∗∗*p* < 0.01, ∗*p* < 0.05. ANOVA followed by Tukey’s multiple comparison was used for the statistical analysis.

To ascertain whether the behavioral phenotype observed in the *T*. *gondii*-infected groups would be reflected by altered synaptic neurotransmission, we employed flow cytometry analysis of isolated synaptosomes (i.e., flow synaptometry)^49^ from the striatum, hippocampus, and cortex. Briefly, this method permits quantification of synaptosome subclasses based on simultaneous fluorescent staining. Specifically, staining for pre- and postsynaptic proteins Synaptophysin and Homer1 were used to select intact synaptosomes. VGLUT1 staining of intact synaptosomes was further used to quantify the relative abundance of glutamatergic synapses (Figures 3A–3C).

Differences in the overall frequency of intact synapses in the striatum, hippocampus, and cortex following *T*. *gondii* infection were not evident (Figure 3D). However, in-depth analysis of the fraction of intact synaptosomes highlighted a significant reduction in the population size of VGLUT1^+^ synaptosomes (i.e., glutamatergic synaptosomes) in the *T*. *gondii* group compared to control within the striatum (F_(2,13)_ = 8.8, *p* = 0.0038; Tukey’s multiple comparison [later not directly specified]: *q* = 4.77, *p* = 0.01) and hippocampus (F_(2,15)_ = 13.73, *p* = 0.0004; *q* = 7.37, *p* = 0.0003). Interestingly, *T*. *gondii* did not affect the abundance of intact glutamatergic synaptosomes in the cortex (F_(2,15)_ = 1.34, *p* = 0.29) (Figure 3E). In addition, we used the median fluorescence intensity (MFI) as a relative measure of overall VGLUT1 protein levels within synapses and showed a decrease in *T*. *gondii* infection when compared to non-infected controls in the striatum (F_(2,13)_ = 8.02, *p* = 0.0054; *q* = 4.42, *p* = 0.02), hippocampus (F_(2,15)_ = 13.94, *p* = 0.0004; *q* = 7.37, *p* = 0003), and cortex (F_(2,15)_ = 18.73, *p* < 0.0001; q = 8.57, *p* < 0.0001) (Figure 3F).

Treatment with sulfadiazine increased the percentage of intact glutamatergic synaptosomes in the hippocampus compared to the *T*. *gondii* group (F_(2,15)_ = 13.73, *p* = 0.0004; q = 4.33, *p* = 0.02), with no significant difference compared to non-infected controls (q = 3.05, *p* = 0.11) (Figure 3E). Similarly, MFI of glutamatergic synaptosomes in hippocampus was increased upon sulfadiazine treatment when compared to the *T*. *gondii* group (F_(2,15)_ = 13.94, *p* = 0.0004; *q* = 4.71, *p* = 0.01) and was not different from the control group (*q* = 2.65, *p* = 0.18) (Figure 3F). In contrast to the hippocampus, a comparison of *T*. *gondii*+Sulfa and *T*. *gondii* groups indicated that sulfadiazine did not affect the abundance of glutamatergic synaptosomes (F_(2,13)_ = 8.8, *p* = 0.0038; *q* = 0.05, *p* = 0.99) (Figure 3E) nor the MFI (F_(2,13)_ = 8.02, *p* = 0.0054; *q* = 0.18, *p* = 0.99) (Figure 3F) in the striatum. Similarly, sulfadiazine did not fully restore the deteriorated MFI in the cortex as revealed by comparisons between *T*. *gondii*+Sulfa vs. *T*. *gondii* (F_(2,15)_ = 18.73, *p* < 0.0001; *q* = 3.27, *p* = 0.085) and control vs. *T*. *gondii*+Sulfa (*q* = 5.31, *p* = 0.0051) group (Figure 3F).

Taken together, flow synaptometry revealed a dysregulation of glutamatergic neurotransmission within the striatum, hippocampus, and cortex of *T*. *gondii*-infected animals. However, this dysregulation differed with respect to the brain area; in the striatum and hippocampus, *T*. *gondii* induced a decrease in the number of intact glutamatergic synapses and the overall level of VGLUT1, whereas in the cortex, *T*. *gondii* only diminished VGLUT1 abundance without affecting glutamatergic synaptic integrity. Anti-parasitic sulfadiazine treatment only restored the VGLUT1 level and glutamatergic synaptic integrity in the hippocampus and had no significant effect within the striatum and cortex.

### Indirect modulation of neuroinflammatory responses and immune dynamics via targeted parasite control by sulfadiazine treatment in cerebral toxoplasmosis

**Figure 4:**
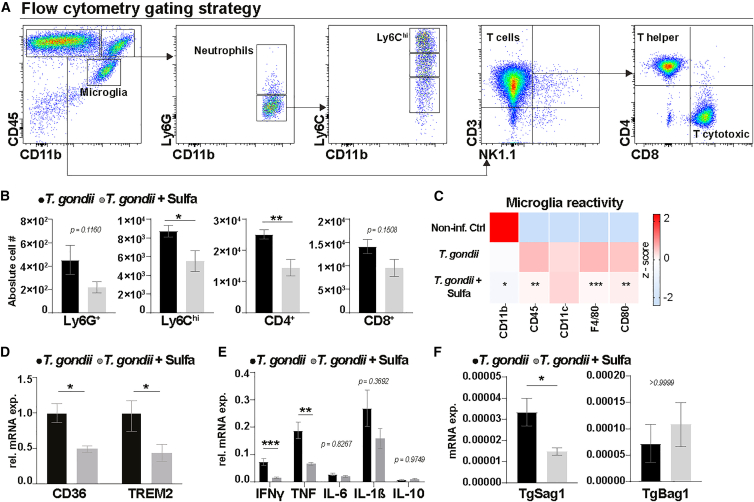
Indirect modulation of neuroinflammatory and immune cell responses through targeted parasite burden reduction by sulfadiazine in chronic *T*. *gondii* infection (A) Representative gating strategy for flow cytometric analysis of mouse brains chronically infected with *T*. *gondii*. Cells isolated from the whole brain tissue were first selected according to their size and granularity in the forward and side light scatterplots (FSC-A, SSC-A, not shown), and only live single cells were gated. Microglia were identified by CD11b^+^CD45^int^ expression, while myeloid cells were first gated on CD11b^+^CD45^high^, then further subdivided into Ly6G^+^ neutrophils and Ly6G^−^ mononuclear cells. Mononuclear cells were further classified based on Ly6C expression. Lymphocytes were identified through CD11b^−^CD45^high^ expression and further distinguished by NK1.1^+^ and CD3^+^markers, with CD3^+^ T cells categorized into CD4^+^ T helper and CD8^+^ cytotoxic subsets. (B) Bar charts show the absolute cell count of peripheral immune cells recruited to the brains of *T*. *gondii* and *T*. *gondii*+Sulfa groups. (C) *Z* score heatmap representing standardized median fluorescence intensity (MFI) values of microglial surface markers across experimental groups. Asterisks denote statistical significance based on raw MFI values, highlighting differential expression following *T*. *gondii* infection and sulfadiazine treatment. (D) Relative mRNA levels of phagocytosis-associated markers CD36 and TREM2, with fold changes normalized to *Hprt* and adjusted to the mean values of the *T*. *gondii*-infected group. (E) Relative mRNA expression levels of cytokines in the brains of infected and treated mice, normalized to *Hprt*. (F) Expression of *T*. *gondii* stage-specific markers in brains of chronically infected mice. SAG1 encodes the major surface antigen of tachyzoites, while BAG1 is specific to the bradyzoite stage, indicating establishment of chronic infection. Expression values are normalized to *Gapdh*. *n* = 5 per group. Statistical analysis: for plots (B, D, E, and F), comparisons between *T*. *gondii*-infected and sulfadiazine-treated groups were performed using the Mann-Whitney U test. For plot (C), data were analyzed by one-way ANOVA followed by Holm-Šidák post hoc test for predefined comparisons between *T*. *gondii*-infected and sulfadiazine-treated groups. Bars represent the mean values ±standard error of the mean (∗*p* < 0.05, ∗∗∗*p* < 0.001, ∗∗*p* < 0.01).

Activated microglia and peripheral immune cells recruited to the CNS triggered by *T*. *gondii* infection engage directly with neuronal circuits and play a critical role in the neuroinflammatory response to cerebral toxoplasmosis.^50^^,^^51^ This direct interaction is essential for understanding how chronic *T*. *gondii* infection reshapes the CNS immune landscape, influencing glutamatergic neurotransmission, synaptic integrity, and behavioral outcomes. To further dissect immune-dependent effects, we conducted a detailed analysis of CNS immune cell composition during the chronic and latent stages of toxoplasmosis to delineate these interactions and their implications for neural functioning (Figure 4A).

Flow cytometric analysis demonstrated a significant recruitment of CD45^+^CD11b^+^ myeloid cells into the CNS in response to *T*. *gondii* infection, while the non-infected control brains lacked immune cell recruitment, consistent with our prior findings.^27^^,^^50^^,^^51^^,^^52^ We observed a significant reduction in the number of Ly6C^hi^ monocytes and CD4^+^ T cells within the brain parenchyma of the sulfadiazine-treated group compared to the infected untreated group. While Ly6G^+^ neutrophils and CD8^+^ T cells also showed a trend toward lower numbers in the treated group, these differences were statistically not significant (Figure 4B).

We then evaluated microglial reactivity by the expression of specific activation markers, including CD11b, CD45, F4/80, and CD80 (Figure 4C). Our findings showed a significant reduction in these markers in the *T*. *gondii*+Sulfa group compared to the untreated *T*. *gondii*-infected group. To complement these findings, we examined changes at the molecular level by measuring the mRNA expression of phagocytic markers CD36 and TREM2 (Figure 4D). Our findings revealed that both CD36 and TREM2 mRNA levels were significantly reduced in the brains of sulfadiazine-treated mice compared to those infected but untreated (Figures 4C and 4D).

After analyzing the composition of immune cells and the activation state of microglia, we examined the cytokine profile to understand the broader effects of changes in immune cell dynamics on the inflammatory environment. We employed semi-quantitative RT-qPCR to assess the expression levels of interferon gamma (IFN-γ), tumor necrosis factor (TNF), interleukin (IL)-6, IL-1β, and IL-10, within the CNS. Our findings revealed a notable decrease in the levels of the pro-inflammatory cytokines IFN-γ and TNF (Figure 4E). This cytokine expression pattern aligns with the observed reduction in immune cells recruitment (Figure 4B) and microglial activity (Figure 4C) and supports a reduction in the neuroinflammatory response following sulfadiazine treatment.

To further investigate whether these changes in the immune environment were linked to a decrease in active parasite load, we conducted semi-quantitative RT-qPCR to assess the expression levels of *Sag1* and *Bag1*, which correspond to active and quiescent parasite stages, respectively. Our results showed a significant reduction in *Sag1* expression, indicating a lower number of active tachyzoites, while *Bag1* levels remained unchanged, suggesting that the dormant form of the parasite was not impacted by sulfadiazine treatment. This pattern supports the hypothesis that sulfadiazine’s reduction of active parasite levels indirectly contributes to the attenuation of the neuroinflammatory response (Figure 4F).

### *T*. *gondii* alters gene expression of markers related to immune response and neuromodulation in the striatum

**Figure 5:**
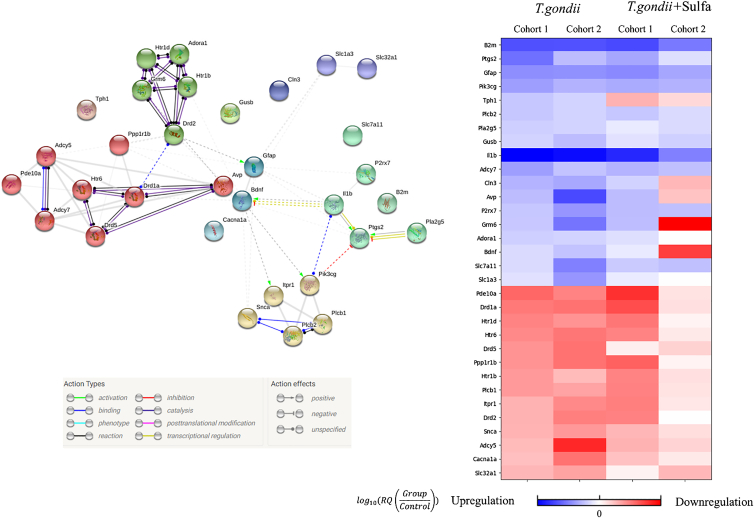
Interaction map of the genes affected by *T*. *gondii* Left panel features functional clusters of proteins encoded by genes affected by *T*. *gondii* infection in the brain and the respective interactions between them (see Action Types and Action effects). The thickness of the line is proportional to the confidence score from STRING database. Genes belonging to the same functional cluster are indicated with the same color. Right panel depicts the gene expression heatmap for two mice cohorts from *T*. *gondii* and *T*. *gondii*+Sulfa groups (see Table S8 for raw RQ values).

Flow-cytometric analysis of glutamatergic neurotransmission revealed structural and functional deteriorations in the striatum, which, in contrast to the cortex and hippocampus, were not restored with sulfadiazine. Therefore, we decided to further focus on the striatum and analyzed the expression of genes involved in neurotransmission (e.g., GABAergic and glutamatergic) and neuromodulation (dopaminergic and serotonergic) (see STAR Methods for more details and Figure 5 for the results).

First, *T*. *gondii* infection triggered a sustained state of inflammation, indicated by the upregulation of general immune response markers (IL-1β, B2m, Ptgs2, Pla2g5, and GFAP). Second, we detected alterations in intracellular signaling pathways, both in a general context (cAMP signaling: Pde10a, Adcy5, and Adcy7) and in specific pathways (inositol signaling: Plcb1, Plcb2, Itpr1, and Pik3cg). Moreover, extracellular signaling was affected, as indicated by alterations in the expression of proteins involved in vesicular transport (Slc7a11 and Snca), corresponding to biased GABA (Slc32a1) and glutamate (Slc1a3) signaling^1^ (see Table S10 for values of up/down regulation). Neuromodulatory systems were altered in an opposite manner. Dopamine (DrD1a, DrD2, and DrD5) and serotonin receptor genes (Htr1d, Htr1b, and Htr6) were downregulated, whereas the expression of the tryptophan hydroxylase gene Tph1 was upregulated. This pattern is consistent with the hypothesis of biased extracellular neuromodulator levels in latent toxoplasmosis.^30^^,^^53^

Overall, *T*. *gondii* affected the expression of the genes related to neuromodulatory systems, intracellular and extracellular signaling pathways, as well as inflammation, while sulfadiazine treatment only partially normalized these alterations.

## Discussion

Chronic infection with the intracellular parasite *T*. *gondii* elicits lasting alterations in the nervous system of the host and is associated with a spectrum of behavioral changes.^10^^,^^12^^,^^16^ In the present study, we linked chronic toxoplasmosis in mice with subsequent alterations in behavior and synaptic transmission. Specifically, we used complementary behavioral tests to discern motor impairments induced by *T*. *gondii* infection-induced inflammation from potential high-order cognitive malfunctions and quantified the abundance of glutamatergic synapses and VGLUT1 levels via flow synaptometry. To contextualize our findings within the scope of neuroinflammation, a subset of the infected mice was subjected to the treatment with the commonly used antiparasitic drug sulfadiazine. The aim was to determine whether mitigating the neuroinflammatory response to *T*. *gondii* infection could restore synaptic function and alleviate behavioral deficits. To this end, we analyzed the gene expression patterns linked to synaptic transmission and neuroinflammatory responses.

### Disrupted high-order cognitive functions and the ameliorating effect of sulfadiazine

Our initial behavioral analysis identified significant motor impairments caused by *T*. *gondii*, including reduced locomotion and compromised motor coordination. The impaired locomotor activity of the *T*. *gondii* group is in accordance with studies reporting reduced locomotion provoked by chronic *T*. *gondii* infection^22^^,^^23^^,^^31^ but contradicts studies that found no difference or even elevated locomotion in chronic toxoplasmosis.^19^^,^^20^^,^^25^ This ambiguity can be, at least partially, explained by the fact that Afonso et al., Boillat et al., and Castano Barrios et al.^19^^,^^20^^,^^25^ used tachyzoites for the infection, whereas Gatkowska et al., Hermes et al., and Acquarone et al.^22^^,^^23^^,^^31^ utilized cysts, as well as another parasite strain, resulting in different degrees of the infection severity.

To further investigate alterations in cognitive processes, we used tests without a demanding motor component and could thus uncover potential cognitive dysfunctions, even in the presence of motor impairments. With the three-chamber SI test, we observed an impaired social novelty response, similarly as shown earlier by Torres et al.^33^ (but see^31^ for contradicting results), whereas the custom-developed behavioral flexibility test allowed us to detect diminished behavioral flexibility in shifting between goal-directed and habitual behavior upon *T*. *gondii* infection. Importantly, our results in diminished behavioral flexibility in shifting between goal-directed and habitual behavior agree with earlier studies suggesting impaired goal-directed behavior upon *T*. *gondii* infection in humans^10^ and rodents^53^^,^^54^ and propose a causality between one of the fundamental disturbances in decision-making disorders, including addiction, depression, OCD,^44^^,^^45^^,^^46^ and chronic toxoplasmosis.

The anti-parasitic drug sulfadiazine was previously shown to ameliorate symptoms of *T*. *gondii* infection in humans as well as animal models of toxoplasmosis.^55^^,^^56^ In our study, treatment of infected mice with sulfadiazine restored the impairments in locomotion (Figure 1B) and the ability to flexibly shift between goal-directed and habitual behavior (Figure 2), which is consistent with recent studies reporting behavioral improvements in mice after the application of anti-inflammatory drugs.^53^^,^^54^ However, sulfadiazine did not fully restore the deficits in the social novelty response (Figure 1A) or motor coordination.

The decrease in active-stage tachyzoite markers (e.g., SAG1), together with stable levels of dormant-stage bradyzoite markers (e.g., BAG1) (Figure 4E), indicates sulfadiazine’s beneficial effects by inhibiting parasite replication and, in turn, reducing the reported neuroinflammatory response (Figures 4B–4E). Therefore, we argue that a partial recovery in the marble burying (Figure 1C) and SI tests (Figure 1A) may be explained by an overall reduced, however, not fully resolved, level of inflammation without complete elimination of *T*. *gondii* by sulfadiazine treatment as demonstrated by Lang et al.^30^ This interpretation is further supported by the persistent dysregulation of genes across multiple neurotransmitter and immune systems despite sulfadiazine treatment (Table S10).

Thus, similarly to the earlier works, our data support *T*. *gondii*-associated neuroinflammation as the cause of the described multi-domain behavioral changes.^20^^,^^54^^,^^57^^,^^58^ However, to shed light on the mechanism of these inflammation-induced behavioral abnormalities, we aimed to further explore how *T*. *gondii*-associated neuroinflammation might affect neurotransmission.

### *T*. *gondii* impairs excitatory transmission in a region-specific manner

Recent studies^53^^,^^54^ revealed decreased expression of key synaptic structural proteins at pre- (synaptophysin) and postsynaptic (PSD95) sites, which was accompanied by lower spine density, as reported previously,^32^ and lower complexity of neuronal morphology after *T*. *gondii* infection, in agreement with our previous reports.^29^ In addition to these structural changes, *T*. *gondii* was reported to interfere at the protein level, with glutamatergic^5^^,^^30^^,^^32^^,^^33^ and GABAergic^34^^,^^35^ systems ultimately shifting excitation-inhibition (i.e., E/I) balance toward excitation, with some studies even reporting excitotoxicity^32^ and seizures.^34^

Thus, the relationship between *T*. *gondii* and neurotransmission may reflect (1) structural changes of neuronal morphology or synapses and (2) molecular changes at the level of neurotransmission-related proteins such as VGLUT1, GABA, NMDAR, and others. Because previous studies primarily assessed protein levels via western blot analysis^32^^,^^33^^,^^53^^,^^54^ or quantifying staining,^34^ they did not directly differentiate between these mechanisms. To circumvent this technical limitation, we took advantage of our recently developed approach to assess single, isolated synapses in high quantities by flow synaptometry^49^ and focused on the glutamatergic neurotransmission.

With flow synaptometry, we were able to detect the integrity of synapses as well as alterations in glutamatergic signaling (via VGLUT1 levels) in intact synaptosomes and revealed impaired glutamatergic signaling in the striatum, hippocampus, and cortex following *T*. *gondii* infection. The overall decrease in VGLUT1 upon *T*. *gondii* infection is in line with reports showing elevated extracellular glutamate levels linked to excitotoxicity, with diminished VGLUT1 levels.^32^ Interestingly, earlier studies have shown that *T*. *gondii* cysts preferentially form in cortical areas,^20^^,^^33^ co-localize with NMDA receptors,^33^ and trigger the production of NMDAR autoantibodies.^59^ Combined with our findings revealing no effect of *T*. *gondii* on the structural integrity of glutamatergic cortical synapses (Figure 3E), these observations point toward the parasite’s capacity to modify glutamatergic signaling in ways that are not due to the general tissue damage but instead may result either from (1) a direct interaction between cysts and synaptic glutamatergic transmission and/or from (2) the host’s neuroinflammatory response. In this context, our flow synaptometry data distinguish structural and functional alterations in synaptic neurotransmission, and having the sulfadiazine-treated group allows us to isolate the contribution of chronic neuroinflammation to these alterations. At the next step, we further elucidated the effects of neuroinflammation on the brain homeostasis in general and neurotransmission in particular.

### Indirect restoration of glutamatergic synaptic transmission through neuroinflammation control by sulfadiazine in a region-specific manner

Extensive prior research,^20^^,^^50^^,^^52^^,^^53^^,^^54^^,^^60^ alongside our current immunological (Figure 4) and transcriptome (Figure 5) data, demonstrate a widespread neuroinflammatory response upon *T*. *gondii* infection, which impacts synaptic transmission and behavior.^20^^,^^53^^,^^54^ In our study, we employed sulfadiazine, an anti-parasitic drug that inhibits the production of *para*-aminobenzoic acid production crucial for the parasite’s folic acid synthesis and DNA replication.^55^ Thus, sulfadiazine can limit peripheral parasite replication, preventing tachyzoites from breaching into the CNS, resulting in fewer brain cysts.^30^ However, to evaluate the effects of sulfadiazine independently of initial parasite burden reduction, treatment was initiated on day 10, allowing for the establishment of a latent infection with a higher number of cysts in the brain. Our findings revealed that sulfadiazine significantly moderates neuroinflammation (Figure 4), as evidenced by reduced microglial activation and the recruitment of myeloid-derived mononuclear cells as well as T lymphocytes into the CNS.

Moreover, the diminished expression of microglial phagocytic markers such as F4/80, CD36, and TREM2 suggests a reduction in their phagocytic activity, which may lead to lower levels of synaptic pruning typically exacerbated by inflammatory triggers. Therefore, controlling neuroinflammation mitigates immediate immune cell reactivity and positively influences long-term synaptic and behavioral outcomes. These findings highlight the potential of focusing on neuroinflammatory pathways to preserve neural integrity and improve outcomes in conditions characterized by chronic inflammation.

Interestingly, sulfadiazine treatment did not affect glutamatergic transmission in the striatum and cortex but restored both structural integrity and presynaptic glutamate levels in the hippocampus (Figure 3). The neuroprotective effect of reduced neuroinflammation observed in the hippocampus is in line with recent studies showing similar effects of other anti-inflammatory drugs on synaptic structures and neuronal morphology.^53^^,^^54^ However, the absence of a treatment effect on synaptic transmission proxies in the striatum suggests that the observed structural and VGLUT1 changes in the striatum may be less dependent on elevated neuroinflammation and more linked to discussed glutamatergic excitotoxicity linked to GLT-1 downregulation^32^ and/or crosstalk between cysts and glutamatergic system.^33^^,^^59^ Alternatively, the striatum may exhibit heightened sensitivity to neuroinflammatory processes. Consequently, (1) the anti-inflammatory effect of sulfadiazine might be insufficient to fully counteract the persistent inflammation, and/or (2) the striatum may require a longer recovery period to restore structural and functional integrity after an inflammation-induced disruption.

Our gene expression analysis of the striatum tissue exposed the consistent upregulation of neuroinflammation markers such as Il1b, B2m, Gfap, and Pik3cg, which was not normalized by anti-inflammatory sulfadiazine treatment (Figure 5; Table S10), suggesting that in both *T*. *gondii* and *T*. *gondii*+Sulfa the neuroinflammation is still present in the striatum. At the same time, study by Brito et al.^57^ showed the regional dependent effect of *T*. *gondii*-sparked neuroinflammation with, for instance, nitric oxide being lower in cortex and striatum, compared to hippocampus. In addition, previous non-Toxoplasma studies^61^^,^^62^^,^^63^ suggest striatum as being more resistant to neuroinflammation-driven deterioration compared to cortex and hippocampus. Thus, these prior findings additionally support the potential regional dependency of *T*. *gondii*-induced neuroinflammation as one of the factors influencing the observed changes in the neurotransmission. Recent findings indicate that the secretion of IFN-γ by a subpopulation of T cells during *T*. *gondii* infection depends on elevated levels of extracellular glutamate in the brain—a feature that was previously reported following *T*. *gondii* infection.^32^^,^^64^ Thus, the final region-specific outcome may reflect a balance between excitotoxic damage and the mixed effects of neuroinflammation: ranging from further deterioration to neuroprotection.

### Limitations of the study

This study focuses on the subgroup VGLUT1, critical for neurotransmission. However, alterations in VGLUT2, which is primarily linked to synaptic plasticity, have been reported in chronic toxoplasmosis.^33^ Another limitation relates to our focus on excitatory neurotransmission, whereas *T*. *gondii* has been shown to affect the metabolism of gamma-aminobutyric acid (GABA)^33^^,^^34^ and directly use it as a carbon source for its metabolism.^65^ As GABAergic inhibitory neurotransmission was not addressed in this study, we limited our interpretation to excitatory neurotransmission.

Since *T*. *gondii* infection causes motor impairment, experimental blindness could not be ensured with respect to the phenotype during the behavioral tests, and this might have unconsciously affected animal handling. In addition, for the behavioral flexibility test, the exact order of testing at the outcome devaluation phase could affect animal performance; the first presented context might provoke a higher performance level compared to the second context, since no reward was present. At the same time, due to severe motor impairments, not all animals from the *T*. *gondii* group could pass the performance criteria (Figure S5) during the behavioral flexibility test and were excluded from further testing. Thus, the size of the *T*. *gondii* group (*n* = 6) was smaller than that of the control (*n* = 13) and *T*. *gondii*+Sulfa (*n* = 13) groups, making the *T*. *gondii* group more susceptible to the above-described behavioral limitations during the behavioral flexibility test. In this regard, we argue that our conclusions are not derived from a single test (e.g., behavioral flexibility) but rather from a battery of complementary behavioral tests, thereby compensating for potential biases in the general outcomes. Another insecurity is the complex pharmacology of Sulfadiazine and its stability that requires the refreshment of the solution. Some of the observed variability concerning the *T.gondii*+Sulfa results presented in Figure 5 may be explained by variations in Sulfadiazine steady-state levels.

### Future directions

Exploring the interaction between excitatory and inhibitory systems in a region-specific manner and its link to behavioral outcomes in individuals infected with *T*. *gondii* presents an intriguing avenue for future research. Previous studies have shown that *T*. *gondii* infection specifically reduces innate anxiety toward predator odor in mice, leading to increased life-threatening risk-taking behavior.^20^^,^^66^^,^^67^ The increased risk-taking behavior to predator odor has been further associated with the regulation of specific GABAergic interneurons in the ventral hippocampus.^68^ The functional dichotomy along the dorsoventral hippocampal axis^69^ was not considered in this study. Investigating this aspect, along with the impact of *T*. *gondii* infection on specific glutamatergic and GABAergic networks in distinct brain regions, is a valuable direction for future research.

Another prospective research direction is to assess chronic *T*. *gondii* infection at the later time points (i.e., weeks or months post-infection). Specifically, McGovern et al. showed that at 20 mpi mice exhibited no signs of neuroinflammation and parasite presence, even though the initial infection with *T*. *gondii* was confirmed by various complementary techniques such as weight loss, seropositivity tests, and the expression of the fluorescent marker triggered by parasite-driven Cre-mediated recombination. Additionally, McGovern et al. found no signs of the impairments in long- and short-term memory at 20 mpi, suggesting that the detected natural clearance of the parasite might have caused the behavioral restoration.^70^ However, this can also be a selective feature of the tested behavior, because the other study from Jung et al. similarly showed no memory deficits at 6 mpi, whereas the *T*. *gondii* was linked with alterations of the host immune response.^71^ Additionally, a study from Ingram et al. showed the persistent loss of the predator aversion with no parasite or brain inflammation detected.^24^ Combined together above described studies^24^^,^^70^^,^^71^ and other research supporting (1) inflammation-driven behavioral changes,^20^^,^^54^^,^^57^^,^^58^ (2) structural brain damage,^29^^,^^32^^,^^53^^,^^54^ and (3) selective functional changes in the neurotransmission^33^^,^^59^ induced by *T*. *gondii* and linked to the behavior call for the future studies assessing both the broad behavior phenotype and neurotransmission changes at the later (e.g., 20 mpi) time point where *T*. *gondii* was reported to be cleared by the host.^70^ In this way, researchers can better characterize behavioral changes that emerge during later stages of chronic infection and determine neurotransmission changes in which brain areas drive these behavior alterations. In this light, our work (1) suggests and tests the optimal method to target neurotransmission changes at both structural and functional level—flow synaptometry and (2) hints toward the regional tropism of the potential changes.

### Conclusion

Our results demonstrate the profound effects of chronic and latent toxoplasmosis on motor and cognitive aspects of mouse behavior. Sulfadiazine treatment was shown to partially restore most of the affected behavioral traits, ameliorating the ongoing neuroinflammation but not eliminating it. In depth analysis of glutamatergic synaptic transmission with a flow synaptometry technique revealed region-specific structural and functional deterioration of glutamatergic neurotransmission, which was not entirely restored by sulfadiazine treatment. We suggest that the revealed region-specific effects of latent toxoplasmosis and sulfadiazine treatment on synaptic transmission may account for the observed treatment-resistant behavioral malfunctions. Thus, further investigation, particularly into region specificity of *T*. *gondii*-induced effects, may pave the way for novel therapy approaches.

## Resource availability

### Lead contact

Requests for further information should be directed to and will be fulfilled by the lead contact, Ildiko Rita Dunay (ildikodunay@gmail.com).

### Materials availability

This study did not generate any unique reagents.

### Data and code availability

• This paper does not report datasets of a standardized data type (e.g., sequencing, proteomics, and crystallography). Custom-formatted data generated for this study can be shared upon request. Additional details on the analysis and experiments can, also, be found in the project related repository https://github.com/IuriiSavvateev/Toxoplasma_behaviour_neurotransmission.
• This paper does not report on the original code. Codes used for the statistical analysis can be shared upon request.
• Graphical abstract and panel 1 of Figure 1 were created in BioRender. Dunay, I. (2025) https://BioRender.com/3vm7knl.

## Acknowledgments

We thank Petra Grüneberg for the exceptional technical assistance and Karl-Heinz Smalla for valuable scientific discussions.

This work was supported by the 10.13039/100004875DFG, DU1112/3-1, and the 10.13039/100030923Leibniz Institute for Neurobiology Magdeburg. The work of Iurii Savvateev was supported by a DAAD scholarship for Master’s studies in Germany.

## Author contributions

I.R.D., D.M., and I.S. conceptualized the study. The investigation was performed by I.S., D.M., L.M., and H.P.D. Formal analysis was performed by I.S., D.M., and L.M. Funding acquisition was provided by I.R.D. Resources were supplied by I.R.D. and D.M. Visualization was performed by I.S. and L.M. Writing was performed by I.S., L.M., and I.R.D. The script was reviewed by J.S., H.P.D., D.M., S.M., and B.H.S.

All authors approved this manuscript and provided consent for publication.

## Declaration of interests

The authors declare no competing interests.

## STAR★Methods

### Key resources table

**Table:** 

REAGENT or RESOURCE	SOURCE	IDENTIFIER
**Antibodies**		

Zombie NIR fixable dye	BioLegend	Cat#423105; RRID:AB_11150976
CD16/32 (clone 93) purified	BioLegend	Cat#101302; RRID:AB_312801
CD45 (clone 30-F11), BV510	BioLegend	Cat#103138; RRID:AB_493715
CD11b (clone M1/70), APC	BioLegend	Cat#101212; RRID:AB_312791
CD11b (clone M1/70), APC-Cy7	BioLegend	Cat#101226; RRID:AB_830642
Ly6G (clone 1A8), BV421	BioLegend	Cat#127627; RRID:AB_11149544
Ly6C (clone HK1.4), PerCP/Cy5.5	BioLegend	Cat#128012; RRID:AB_893228
CD11c (clone N418), PE-Cy7	BioLegend	Cat#117318; RRID:AB_1134238
F4/80 (clone BM8), FITC	BioLegend	Cat#123107; RRID:AB_893492
CD80 (clone 16-10A1), FITC	BioLegend	Cat#104705; RRID:AB_313390
CD3 (clone 17A2), PE	BioLegend	Cat#100205; RRID:AB_312660
CD4 (clone GK1.5), FITC	BioLegend	Cat#100405; RRID:AB_312707
CD8 (clone 53-6.7), APC	BioLegend	Cat#100711; RRID:AB_312753
NK1.1 (clone PK136), PE-Cy7	BioLegend	Cat#108713; RRID:AB_313538
Gephyrin	Abcam	Cat#AB136343
Homer1	R&D Systems	Cat#MAB6889; RRID: AB_10889999
Synaptophysin	Synaptic Systems	Cat#101004; RRID: AB_1210382
VGLUT1	Synaptic Systems	Cat#135303; RRID: AB_887875
Goat anti-Mouse AlexaFluor™ 405	Thermo Fisher Scientific	Cat#A-31553; RRID: AB_221604
Goat anti-Rabbit AlexaFluor® 488	Abcam	Cat#ab150081; RRID: AB_2734747)
Goat anti-Chicken AlexaFluor™ 546	Thermo Fisher Scientific	Cat#A-11040; RRID: AB_2534097
Goat anti-Guinea Pig AlexaFluor™ 647	Thermo Fisher Scientific	Cat#A-21450; RRID: AB_2535867

**Commercial Assays**		

Power SYBR Green RNA-to-CT 1-Step Kit	Thermo Fisher Scientific	Cat#4389986
TaqMan™ RNA-to-CT™ 1-Step Kit	Thermo Fisher Scientific	Cat#4392938
Foxp3/Transcription Factor Staining Buffer Set	Thermo Fisher Scientific	Cat#00552300
GABA and Glutamate	Qiagen	Cat#330231 PAMM-158ZA
Dopamine and Serotonin	Qiagen	Cat#330231 PAMM-152ZA
RT2 SYBR green mastermix	Qiagen	Cat#330500
Quick-DNA/RNA Miniprep Kit	Zymo Research	Cat#D7005

**Chemicals, peptides, and recombinant proteins**		

RNAlater™	Thermo Fisher Scientific	Cat#AM7020
TRIzol™ Reagent	Thermo Fisher Scientific	Cat#15596026
DPBS, calcium, magnesium	Thermo Fisher Scientific	Cat#14040117
HBSS	Thermo Fisher Scientific	Cat#14170112
HEPES (1M)	Thermo Fisher Scientific	Cat#15630056
Percoll	Cytiva	Cat#17089101
sulfadiazine	Sigma-Aldrich	S8626-25G
FM4-64	Thermo Fisher Scientific	T13320
DiagNano™ Red Fluorescent Silica Particles, 300 nm	Creative Diagnostics	DNG-L020
DiagNano™ Red Fluorescent Silica Particles, 1 μm	Creative Diagnostics	DNG-L026

**Experimental models: Organisms/strains**		

Mouse: C57BL/6JRj female mice older than 8 weeks	Janvier Laboratories	Cat#SC-C57J-F; RRID: IMSR_RJ:C57BL-6JRJ
*T*. *gondii*, type II ME 49 strain	Liesenfeld et al.^72^	N/A

**Software and algorithms**		

LightCycler 96 software v1.1	Roche	RRID:SCR_012155
FlowJo Software v10.5.3	Becton, Dickinson and Company	RRID:SCR_008520
GraphPad Prism (v9)	GraphPad Software	RRID:SCR_002798
VideoMot2 software	TSE Systems GmbH, Bad Homburg, Germany	N/A

**Oligonucleotides**		

TaqMan Gene Expression Assay *Ifng*	Thermo Fisher Scientific	Mm01168134_m1
TaqMan Gene Expression Assay *Tnf*	Thermo Fisher Scientific	Mm00443258_m1
TaqMan Gene Expression Assay *Il6*	Thermo Fisher Scientific	Mm00446190_m1
TaqMan Gene Expression Assay *Il1b*	Thermo Fisher Scientific	Mm00434228_m1
TaqMan Gene Expression Assay *Il10*	Thermo Fisher Scientific	Mm00439614_m1
TaqMan Gene Expression Assay *Cd36*	Thermo Fisher Scientific	Mm00432403_m1
TaqMan Gene Expression Assay *Trem2*	Thermo Fisher Scientific	Mm04209424_g1
TaqMan Gene Expression Assay *Hprt*	Thermo Fisher Scientific	Mm00446968_m1
SYBR Green Primer *Sag1* forward (*T*. *gondii*): 5′-ATCGCCTGAGAAGCATCACTG	(Fux et al.^73^)	N/A
SYBR Green Primer *Sag1* reverse: 5′-CGAAAATGGAAACGTGACTGG	(Fux et al.^73^)	N/A
SYBR Green Primer *Bag1* forward: 5′-GACGTGGAGTTCGACAGCAAA	(Fux et al.^73^)	N/A
SYBR Green Primer *Bag1* reverse: 5′-ATGGCTCCGTTGTCGACTTCT	(Fux et al.^73^)	N/A

**Other**		

NanoDrop 1000 spectrophotometer	Thermo Fisher Scientific	RRID:SCR_016517
LightCycler 96 System	Roche	Cat#05815916001
Attune NxT Flow Cytometer	Thermo Fisher Scientific	RRID:SCR_019590
5 Hole Nose Poke	TSE Systems	N/A
Real-Time PCR machine	Applied Biosystems 7300	N/A

### Experimental models and subject details

#### Animals

All experiments were performed using female C57BL/6JRj mice (8–15 weeks old; Janvier Laboratories, Saint Berthevin, France). Animals were housed individually with 12-h light/dark cycle and *ad libitum* food, according to institutional guidelines approved by the Animal Studies Committee of Saxony-Anhalt (42502-2-1442): IMMB-01-1089-11. Access to water was free for the entire period, except for the behavioral flexibility testing period. During the behavioral flexibility assessment, animals were water-deprived for approximately 12 h prior to the experiment.

#### *T*. *gondii* infection

Infection was conducted as previously described.^30^^,^^51^ *T*. *gondii* tissue cysts from ME49 type II strain were harvested from the brains of chronically (6–12 months) infected NMRI mice. The brain of an infected NMRI mouse was isolated and homogenized in PBS solution using mortar and pestle before counting the number of cysts using a bright-field microscope at 20× magnification. Each animal in the *T*. *gondii* and *T*. *gondii*+Sulfa groups was injected intraperitoneally (i.p.) with two cysts, whereas the control group received PBS only. From day 10 until the end of the last behavioral test, the *T*. *gondii*+Sulfa group received sulfadiazine treatment via drinking water (400 mg/L), as previously described.^30^

### Method details

#### Behavioral assessments

All animals were subjected to the behavioral paradigms described once before and once after the infection/treatment. The reported results are for the post-infection phase. Behavioral experiments were started 5 weeks after *T*. *gondii* infection to ensure that the infection had become latent by the time of behavioral testing. Animals underwent a series of consecutive established tests (Social Interaction, Open Field, Marble burying, Induced grooming) followed by the behavioral flexibility test. Upon completion of all behavioral assessments, the mice were sacrificed, and their brains were isolated and used for RT-qPCR. For flow synaptometry and flow cytometry experiments, a separate cohort of animals was used, which did not undergo behavioral experiments.

#### Social interaction

The experiment was conducted in a three-compartment box with two side compartments equipped with wire cups and an empty central compartment, as previously described.^36^^,^^74^ Briefly, during three test phases (10 min each), the mouse could explore all compartments. In phase 1, the mouse was alone. In phase 2, an unfamiliar C57BL/6JRj mouse (same sex, same age, stranger 1) was placed in one of the wire cups. In phase 3, another unfamiliar C57BL/6JRj mouse (same sex and age, stranger 2) was placed in the other cup. Time spent in each compartment, time during social contact (sniffing of a stranger by the studied mouse), number of visits to a social interaction area (Figure S8), and transitions between compartments were recorded and analyzed using a computer-assisted tracking system that monitored the position of the studied animal and its moving direction extracted from the relative positions of the head and tail (VideoMot2 software).

#### Open field

In the Open Field test (Figure 1B1), the innate tendency of mice to explore a novel environment is investigated. By analyzing the way an animal explores unfamiliar surroundings, its general activity and exploratory behavior can be assessed, ultimately providing information about motor capabilities, anxiety, and exploration strategies.^38^

The mice were individually placed in the center of an activity box (Figure S9), and allowed to explore freely for 15 min. The animals’ behavior was videotaped from the top. The following parameters were extracted from the video via computer-assisted interface (VideoMot2 software): (i) the relative time (in percent) spent in the central area (center), the area close to the walls (out middle) and in the corners (Figure S9) for the total test duration (15 min) (ii) and for each 5 min interval: 0-5min, 5-10min, 10–15 min, (iii) the path length traveled by the mouse during the test. The analysis and the experiment were conducted, as described previously.^38^^,^^75^

#### Marble burying

A polycarbonate rat cage was filled with mouse bedding material and glass toy marbles were placed on the bedding surface). An experimental mouse was placed in a cage and left undisturbed for 30 min. The (Figure S10) performance of the animal during the test was videotaped from the top. Marbles have been demonstrated to be non-aversive to mice,^76^ suggesting that mice do not deliberately bury marbles in the process of digging into the bedding.^40^ Thus, by analyzing the number of marbles buried, we assessed the natural and spontaneous digging behavior. From the analysis of the video records the following parameters were extracted and calculated: (i) total digging time, (ii) number of digging bouts. The bouts were considered separate if the animal moved to another place in the cage, or at least 6 s hiatus occurred, and (iii) average bout duration: total digging time divided by the number of bouts.

#### Induced grooming

Self-Grooming (for brevity, grooming is used) is a robust behavior in rodent species that occupies up to 30–50% of the wake time.^77^ Two types of grooming can be distinguished: spontaneous (stress-evoked) and artificial grooming (for review, see^43^). Both types are considered maintenance behaviors and are primarily aimed at fur cleaning and thermoregulation^78^ (Spontaneous grooming is evoked by exposing an animal to stress factors as novelty, predator, odor etc., whereas artificial grooming is caused by direct contamination of the animal’s fur (smear with food, mist with water etc.).

For grooming assessment, animals were misted with pure water room temperature (approximately 21°C), as previously described^42^^,^^79^ and subsequently put into the activity box (the same as described for OF test) allowing 10 min free exploration. Behavior was recorded from all four sides (Figure S11) and from the top. The scale system based on Figure S12 was used for the microstructure assessment: no grooming (0), paw licking (1), nose/face/head wash (2), body grooming (3), leg licking (4) and tail/genitals grooming (5). “Correct” transitions include one of the following transitions (0)-(1), (1)-(2), (2)-(3), (3)-(4), (4)-(5). Other transitions were considered as “incorrect”.^42^ The video records were analyzed, and the following parameters were derived as proposed by.^42^ Cumulative measures: (i) total grooming time, (ii) number of grooming bouts (bouts were considered separate if at least 6 s hiatus occurred or the mouse moved to another place in the activity box), and (iii) latency to onset. Characteristics of the grooming microstructure: (i) number of transitions, (ii) percentage of correct transitions, (iii) regional distribution.^42^

#### Behavioral flexibility

The behavioral flexibility test assesses this ability in mice by assessing the context-driven shifting between goal-directed vs. habitual action control in a within-subject instrumental paradigm. Specifically, we adapted a previously described lever-pressing paradigm published by^47^ to “5 Hole Nose Poke” apparatus (TSE Systems).^80^^,^^81^

Mice were water deprived for approximately 12h prior to the experiment and water (10 μL) was used as a reward. Random Ratio (RR) and Random Interval (RI) training schedules, facilitating the development of goal-directed and habitual behavior, respectively, were paired with two different contextual cues: gray side walls or black and white striped side walls. Mice were trained consecutively on the same day to nose poke under RR and RI regimes. Details of the training schedule are listed in Table S8. The devaluation procedure conducted at the end of the training serves to demonstrate the rigidity of habitual behavior and the pliability of goal-directed performance with respect to the changed experimental conditions.^47^The analyzed results include the following parameters obtained during the training: (i) overall poke rates (pokes/min) into an action hole and water dispenser (Figure S13), used to assess the magnitude of the acquired poking behavior and general activity respectively, (ii) number of rewarded (correct according to the schedule) and non-rewarded (not essential for the test completion) pokes into the action hole, potentially characterizing the context-dependent segregation between goal-directed and habitual behavior, (iii) number of non-rewarded pokes into the action hole during the devaluation phase, exposing the rigidity or flexibility of the behavior to the reward devaluation. Normalization of poke rates during the devaluation phase was performed similarly as in ^47^ for lever presses: the poke rate for Valued or Devalued states was divided by the sum of poke rates for Valued and Devalued states.

#### Whole-brain gene expression analysis of immune markers and *T*. *gondii*-specific proteins

Brain tissues were isolated as previously described.^50^^,^^82^^,^^83^ Prior to brain collection, the mice were perfused transcardially with 60 mL ice-cold PBS (with Ca^2+^ and Mg^2+^). The brains were immediately transferred to RNAlater, stored at 4 °C for 24 h, and then stored at −20 °C until further RNA isolation. RNA was isolated using a Quick-DNA/RNA Miniprep kit (Zymo Research, Germany), according to the manufacturer’s instructions.

For cytokine RT-qPCR, TaqMan Gene Assay was used with 300 ng of total RNA in a reaction volume of 10 μL. Triplicate reactions were performed using a LightCycler 96 (Roche). Reverse transcription was performed for 15 min at 48 °C followed by 10 min at 95 °C. Subsequently, 45 amplification cycles were performed, comprising denaturation at 95 °C for 15 s and annealing/elongation at 60 °C for 1 min. Relative gene expression of *Ifng*, *Tnf*, *Il6*, *Il1b*, *Il10*, *Cd36*, and *Trem2* was calculated by normalizing to the gene expression of *Hprt*. Target/reference ratios were calculated using Light-Cycler 96 software (v1.1).

To detect the stage conversion of *T*. *gondii*, RT-qPCR for the mRNA expression levels of *Sag1* and *Bag1* was performed as described by.^73^ Power SYBR Green RNA-to-CT 1-Step Kit was used with 30 ng total RNA in a reaction volume of 10 μL. The amplification profile used was as follows: reverse transcription at 48°C/30 min followed by 10 min at 95 °C, 55 cycles consisting of 95 °C for 15 s, 60 °C for 1 min, 95 °C for 5 s, and 60 °C for 1 min. *Gapdh* mRNA expression levels were chosen as reference and relative target gene mRNA levels were determined by the target gene/reference ratio. Melting curve analysis was used to assess the primer specificity.

#### Screening analysis of gene-expression

Mice were deeply anaesthethized with isoflurane, brains were collected and the striatum was isolated. After dissection, the tissues were immediately frozen in liquid nitrogen. For RNA isolation, striatal tissues from four animals were pooled for each experimental group. The following steps of total RNA isolation were performed using the RNeasy total RNA kit (Qiagen, Cat No./ID: 74104) according to manufacturer’s instructions and RNA was stored at −80°C.

First strand cDNA synthesis was done using RT2 First Strand kit (Qiagen, Cat No./ID: 330401) according to the manufacturer’s instructions with reverse transcription for 15 min at 42°C and inactivation for 5 min at 95°C. SYBR Green-based RT-qPCR used gene arrays “GABA and Glutamate” and “Dopamine and Serotonin” (Qiagen) and RT2 SYBR green mastermix (Qiagen) performed on an Real-Time PCR machine (Applied Biosystems 7300) with 40 amplification cycles consisting of denaturation at 95 °C for 15 s and annealing/elongation at 60 °C for 1 min.

Based on the relative quantification values, genes whose expression was altered by *T*. *gondii* infection were grouped together. To estimate the types of systems that might be affected by changes in gene expression, all genes discussed were placed in functional groups according to the National Institutes of Health (NIH) U.S. National Library of Medicine and National Center for Biotechnology Information (NCBI) Gene Library. To assess the functional clusters of the proteins coded by the listed genes, STRING v 10.0 (Search Tool for the Retrieval of Interacting Genes/Proteins) biological database was used to perform functional clustering.^84^

#### Flow synaptometry

Synaptosomes were obtained from anterior frontal cortex, striatum and hippocampal formation^85^ and labeled for flow synaptometry as described.^49^ In brief, animals were perfused transcardially with 60 mL ice-cold PBS (with Ca^2+^ and Mg^2+^), brains were isolated and dissected into the cortex hippocampus and striatum, snap-frozen in liquid nitrogen, and stored at −80°C until further use. To isolate crude synaptosomes, frozen tissue samples were thawed in buffer A (10 mM HEPES, 320 mM sucrose, protease inhibitors, pH 7.4) and then homogenized. Samples were centrifuged at 1,000 × *g* for 10 min, and the supernatant was centrifuged again at 12,000 × *g* for 20 min. The pellet containing crude membrane fraction was loaded onto a discontinuous sucrose gradient with 0.32 M, 1.0 M, and 1.2 M sucrose layers and centrifuged at 80,000 x *g* at 4 °C for 2h. Synaptosomes were collected from the 1.0/1.2 M sucrose interphase, resuspended in SET buffer (0.32 M sucrose, 1 mM EDTA, 5 mM Tris, pH 7.4) and centrifuged at 100,000 × *g* at 4 °C for 1h. The resulting pellet, containing synaptosomes, was resuspended in SET buffer containing 5% dimethyl sulfoxide, aliquoted, slowly frozen at −80 °C using an isopropanol freezing container, and stored until further use.

For subsequent analysis, synaptosomes were thawed in a water bath at 37 °C and fixed in fixation buffer (FoxP3 transcription factor staining buffer set, Thermo Fisher Scientific). Subsequently, samples were washed using permeabilization buffer (FoxP3 transcription factor staining buffer set) reconstituted with 10% normal goat serum (NGS) before centrifugation at 14,000 × *g at* 4 °C for 10 min. Supernatant was gently removed using a microliter pipette and samples were resuspended in permeabilization puffer (FoxP3 transcription factor staining buffer set) with 10% NGS and stained with primary antibodies against Gephyrin (Abcam), Homer1 (R&D Systems), Synaptophysin (Synaptic Systems), and VGLUT1 (Synaptic Systems). Following incubation, samples were washed and stained with matching secondary antibodies: goat anti-mouse Alexa Fluor 405 (Thermo Fisher Scientific), goat anti-rabbit Alexa Fluor 488 (Abcam), goat anti-chicken Alexa Fluor 546 (Thermo Fisher Scientific), and goat anti-Guinea pig Alexa Fluor 647 (Thermo Fisher Scientific). Finally, samples were washed once more and then resuspended in SET buffer with styryl dye FM4-64 (Thermo Fisher Scientific). Measurements were performed using an Attune NxT flow cytometer (Thermo Fisher Scientific). FSC-triggered detection was replaced by a fluorescence-triggered detection with FM4-64 in the BL3 channel (threshold set to 0.3 × 10^3^ to select only FM4-64-positive events), the event rate was kept below 300 events/s and a size range from 300 to 1,000 nm was applied to detected events in the FSC channel using red fluorescent silica beads with a diameter of 300 nm (Creative Diagnostics) and 1,000 nm (Creative Diagnostics). Fluorescence minus one (FMO) controls were used to determine the level of autofluorescence.

#### Flow cytometry

The brains were collected and immune cells were isolated from freshly obtained hemispheres by mincing and homogenizing the tissue in dissection buffer (1× HBSS; 0.68% Glucose; 13 mM HEPES) using a glass potter following the exact method described elsewhere.^27^^,^^49^^,^^60^^,^^86^ The suspension was strained through a 70-μm cell strainer and centrifuged at 400 × *g* at 4 °C for 20 min. Thereafter, the cell pellet was resuspended in 10 mL 70% isotonic Percoll and then layered with 10 mL 30% isotonic Percoll and 5 mL sterile PBS. The discontinuous Percoll gradient was centrifuged without brake at 800 × *g* at 4 °C for 45 min. Next, the cells residing between the 70% and 30% Percoll layers were collected and washed in 40 mL PBS at 400 × *g* at 4 °C for 10 min. After a second washing step in 5 mL FACS buffer (PBS containing 2% fetal bovine serum and 0.1% sodium azide), the cells were counted in a Neubauer counting chamber and subsequently used for flow cytometry.

For flow cytometric analysis of cell phenotypes, freshly isolated cells were first incubated with Zombie NIR fixable dye (BioLegend) for live/dead discrimination and with an anti-FcγIII/II receptor antibody (CD16/32) to prevent non-specific binding of antibodies. Cells were stained with the following fluorochrome-conjugated antibodies against cell surface markers and incubated for 30 min in FACS buffer: Brilliant Violet 510-CD45, APC-CD11b or APC-Cy7-CD11b, Brilliant Violet 421-Ly6G, PerCP/Cy5.5-Ly6C, PE-Cy7-CD11c, FITC-F4/80, PE-CD3, FITC-CD4, APC-CD8, PE-Cy7-NK1.1. Cells were acquired using an Attune NxT flow cytometer (Thermo Fisher Scientific), and data were analyzed using FlowJo software (version 10.5.3). FMO controls were used to determine autofluorescence levels.

### Quantification and statistical analysis

Statistical analyses for behavioral data were performed using custom-made scripts with the following software packages: Excel (Microsoft) with Real Statistics resource pack (release 5.4),^87^ MATLAB (MathWorks), and Python (release 3.7). Flow Cytometry and Flow Synaptometry data were analyzed with GraphPad Prism (v9). All data are presented as mean ± standard error of the mean (SEM). For all the analyses, the α level for statistical significance was set at 0.05. Datasets were tested for normality using the Shapiro-Wilk test and homoscedasticity (variance homogeneity) using Levene’s test, and an appropriate statistical test was chosen based on Table S11.
