# *Toxoplasma gondii*-induced region-specific disintegration in glutamatergic neurotransmission is linked to cognitive impairments in mice

**DOI:** 10.1016/j.isci.2026.115170

**Published:** 2026-03-06

**Authors:** Iurii Savvateev, Lorena Morton, Henning Peter Düsedau, Johannes Steffen, Sanja Mikulovic, Dirk Montag, Björn H. Schott, Ildiko Rita Dunay

## Main text

(iScience *29*, 114415; January 16, 2026)

In the original publication, first author Iurii Savvateev’s name was misspelled as “Iurii Savaateev.” This has now been corrected. The authors apologize for this error.

