# Supplementary material for: Toxoplasma gondii-induced region-specific disintegration in glutamatergic neurotransmission is linked to cognitive impairments in mice
Source: iScience. 2025 Dec 18;29(1):114415. doi: 10.1016/j.isci.2025.114415 (PMC12818283; doi:10.1016/j.isci.2025.114415)
Supplement: Document S1. Figures S1–S13 and Tables S1–S11 [file mmc1.pdf]

iScience, Volume *29*

Supplemental information

Toxoplasma gondii-induced region-specific disintegration in glutamatergic neurotransmission is linked to cognitive impairments in mice

Iurii Savvateev, Lorena Morton, Henning Peter Düsedau, Johannes Steffen, Sanja Mikulovic, Dirk Montag, Björn H. Schott, and Ildiko Rita Dunay

iScience, Volume ■ ■

Supplemental information

Toxoplasma gondii-induced region-specific disintegration in glutamatergic neurotransmission is linked to cognitive impairments in mice

Iurii Savaateev, Lorena Morton, Henning Peter Düsedau, Johannes Steffen, Sanja Mikulovic, Dirk Montag, Björn H. Schott, and Ildiko Rita Dunay

Supplemental Information

Figures S1-S13
Tables S1-S11

Figures

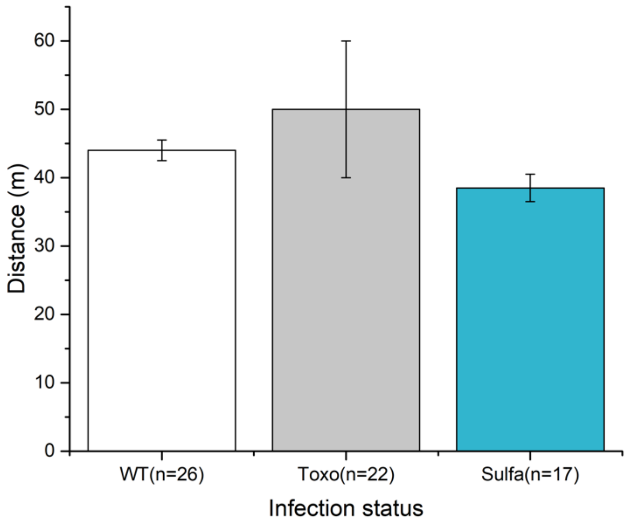

Fig S1. Distance traveled by the animals during Social Interaction test. Kruskal-Wallis test revealed no statistically significant difference ($\chi^2(2)=4.48$, p=0.11) between the distance traveled by Controls (44m±1.5m), *T. gondii* (50m±10m) and *T. gondii*+ Sulfa (38.5m±2m). The bars represent the average distance [m] traveled by the corresponding group±SEM.

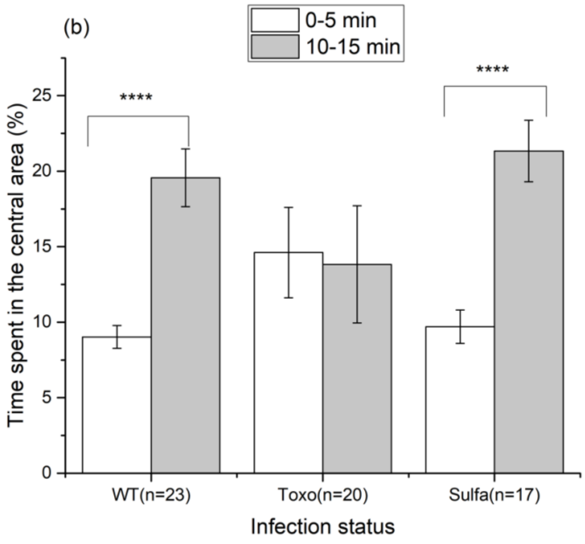

Fig S2. Comparison of the time spent in the central area during the OF test between the first (0-5 min) and the last (10-15 min) intervals for each experimental group (****P<0.0001).

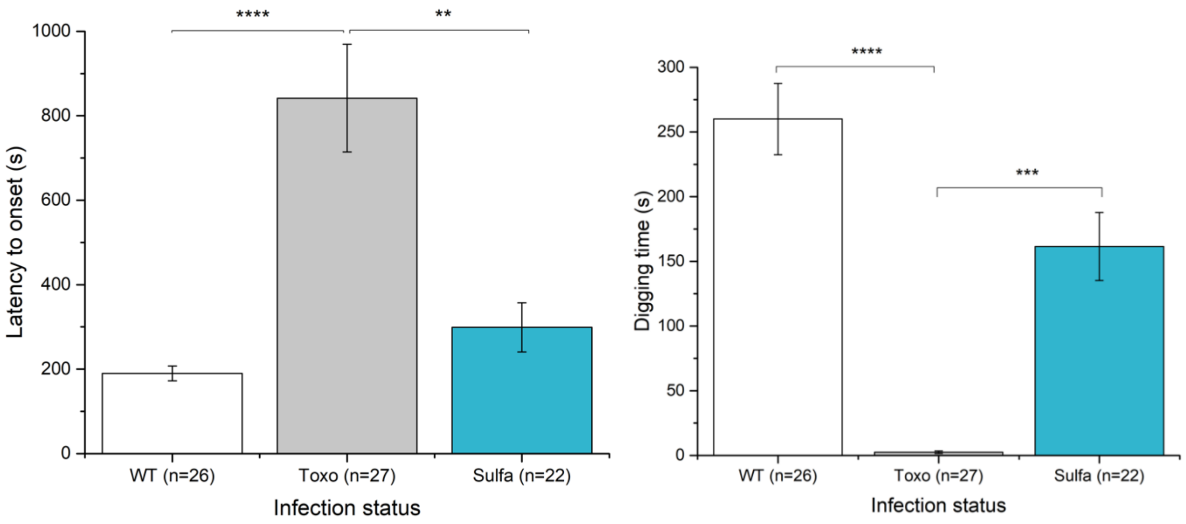

Fig S3. Digging activity. Bar charts feature the parameters of the digging activity measured for each experimental group:latency to onset **(a)** and total digging time **(b)**. Mean values±SEM are shown (*P<0.5,**P<0.01,***P<0.001,****P<0.0001).

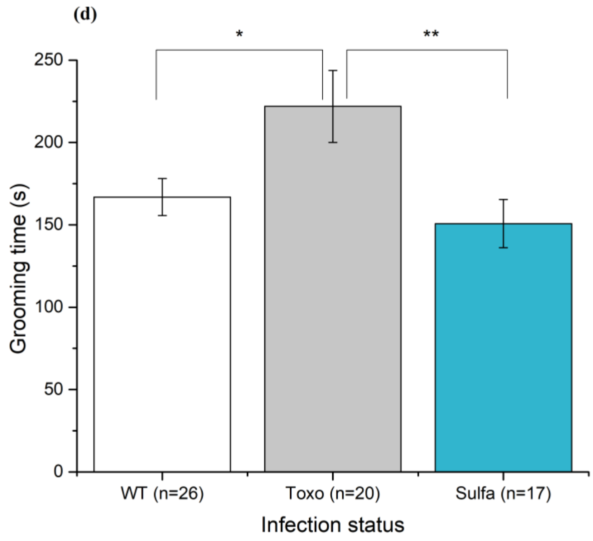

Fig S4. Grooming activity. The total grooming time (s) for each experimental group is shown. Mean values±SEM are shown (*P<0.5,**P<0.01).

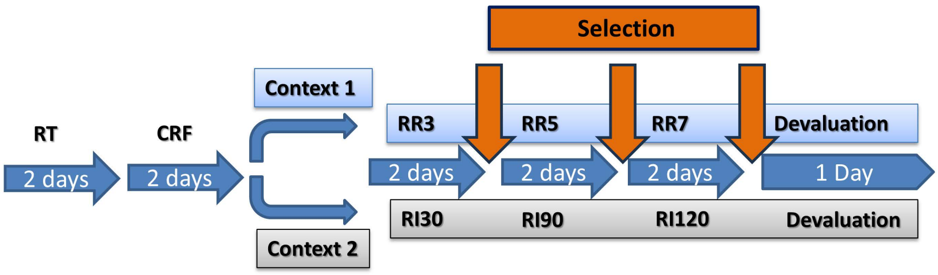

Fig S5. Workflow of the behavior flexibility test

Figure illustrating the procedure of the behavior flexibility test in a chronological order. Arrows depict the number of training days per each phase. RR and RI schedules were used consecutively on the same day of training. **RT schedule**: the nose poke into the hole with the water dispenser results in the delivery of the water drop (~10 µl). **CRF schedule**: in order to receive the water in the hole №6 the mouse should poke into hole №3 first. **RT schedule** was used to familiarize the mice with the position of the water

dispenser and demonstrate the coherence of the Light №6 and water delivery (see *Table №1*). **CRF schedule** aimed to establish the causative link between poking into hole №3 and receiving the reward in the hole №6. **Random Ratio (RR)**: the reinforcement follows on average every 3rd (RR3), 5th (RR5), or 7th (RR7) poke into hole №3, **Random Interval (RI)**: the reward is delivered after on average 30 (RI30), 90 (RI90), 120 (RI) seconds after the first poke into hole №3. **Devaluation test:** mice are not water deprived and the pokes into hole №3 do not result in reward delivery. **Selection:** At the end of each phase, animals that got less than 6 rewards in at least one of the training schedules were excluded from the further training.

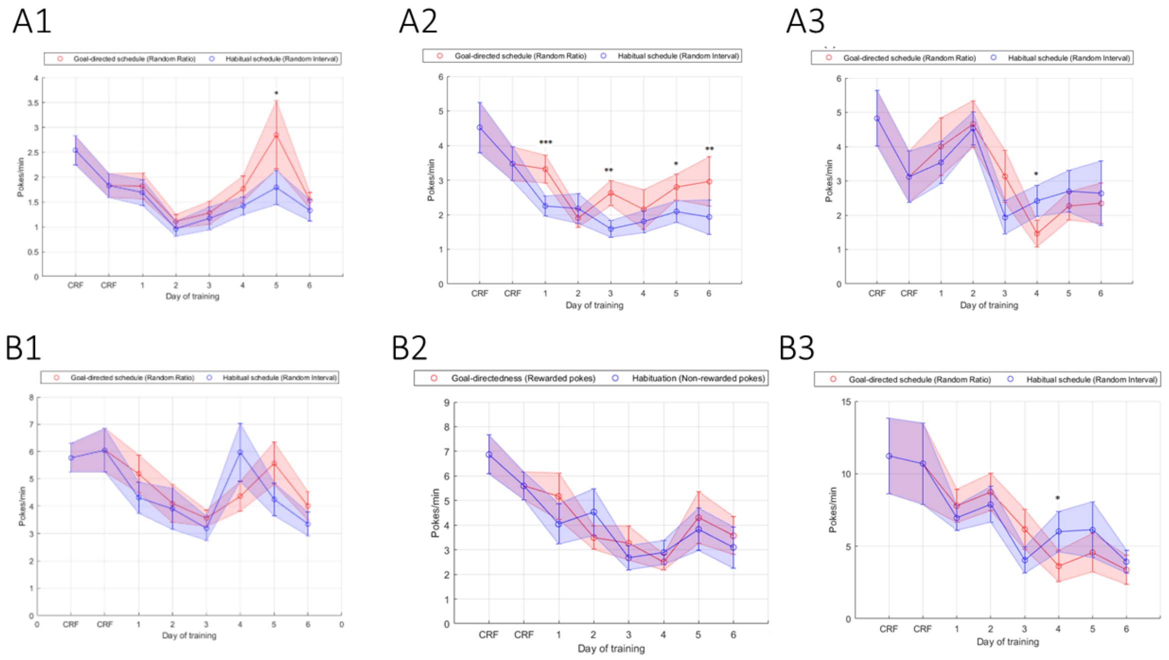

Fig S6. Comparison of the general activity and performance intensity between the reinforcement schedules. Figure depicts the performance intensity **(A1-A3)** and general activity **(B1-B3)** for goal-directed (red) and habitual (blue) training schedules across all days of training for all experimental groups. (A1,B1) –WT, (A2,B2)-Sulfa, (A3,C3)-Toxo. Each point represents the corresponding mean value±SEM (*P<0.5,**P<0.01,***P<0.001,****P<0.0001).*CRF training was done only in one context (see Materials and Methods), but is still shown on the present figure to keep the same format with the previous charts.

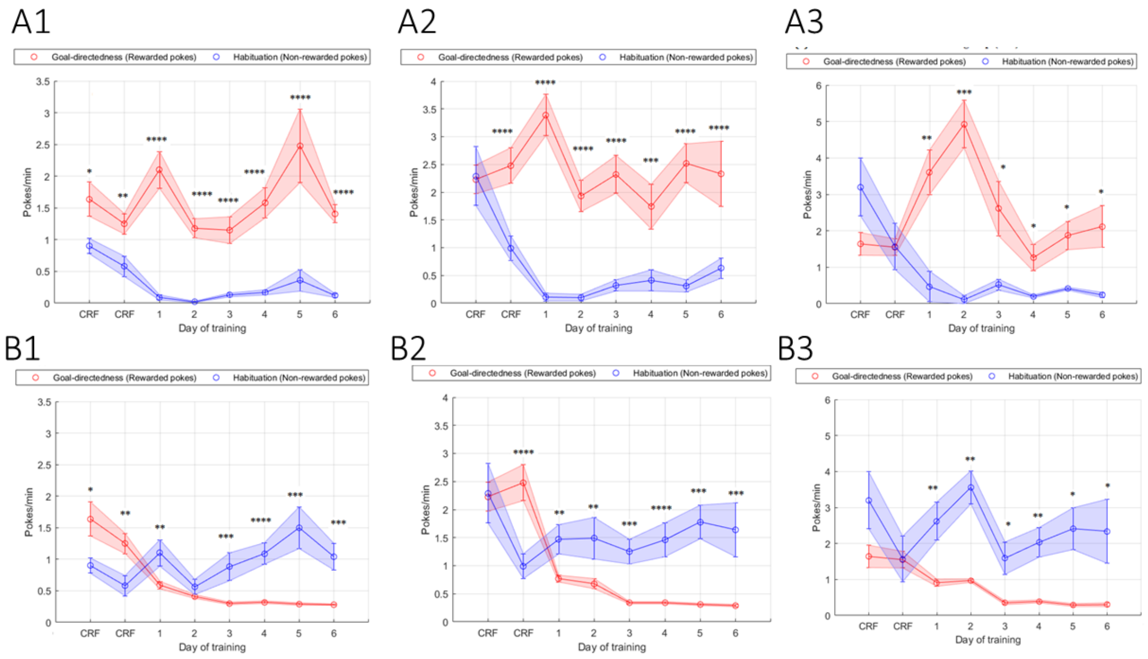

Fig S7. Strategy choice under the goal-directed (RR) and habitual (RI) reinforcement schedule. Each figure shows the poke rate (pokes/min) for rewarded (red color) and non-rewarded pokes (blue color) as a function of the training day. **(A1-A3)** shows data for WT (Control) group, Sulfa group and Toxo group for RR schedule, (**B1-B3**) depicts data for WT (Control) group, Sulfa group and Toxo group for RI schedule. Rewarded pokes serve as a measure of goal-directedness and non-rewarded pokes stand for the habituation degree. Mean values are shown±SEM. Stars correspond to the statistical results of the paired comparisons of rewarded and non-rewarded pokes done for each day. (*P<0.5, **P<0.01, ***P<0.001, ****P<0.0001)

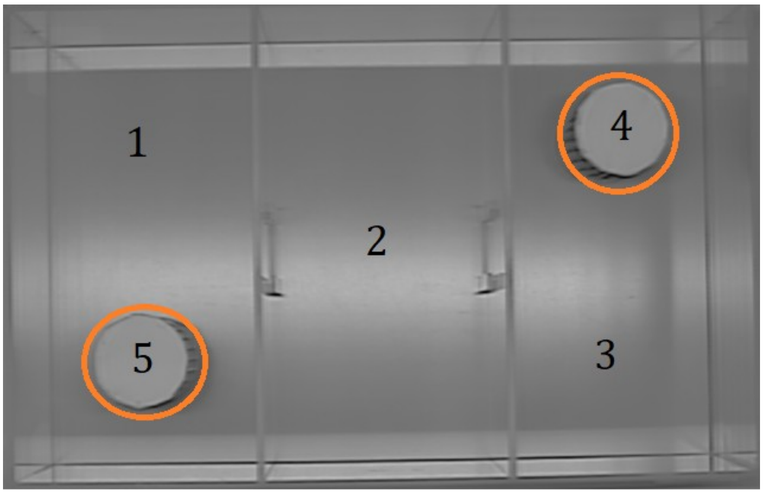

Fig S8. Three compartment box for the social interaction test (view from the top). 1,3 – Side compartments; 2- Central compartment; 4,5- Wire cups. Orange circles features the social interaction zone

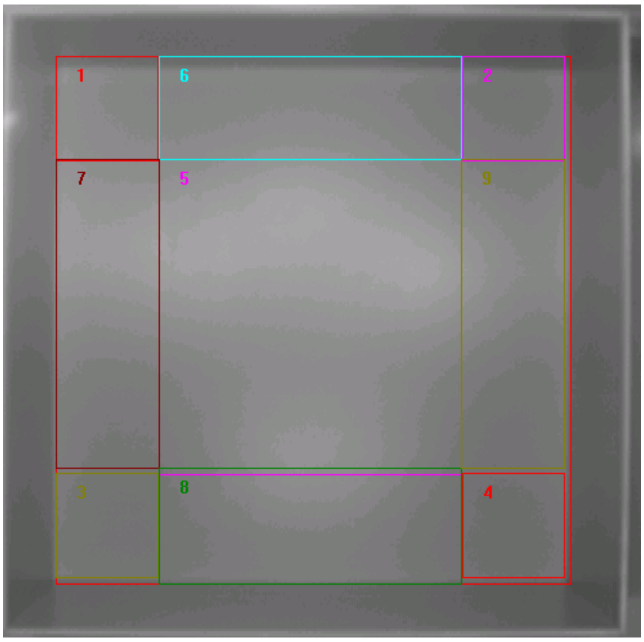

Fig S9. Open field behavioral test. The test is conducted in 50x50 cm square grey plastic arena with grey opaque walls (25 cm height). Illuminance is adjusted to approximately 200 lux. Numbers refer to the different not overlapping parts of the arena: corners (1,2,3,4), out middle (6,7,8,9), center (5). Corners are 10x10 cm each, center is 30x30 cm, and out middle comprises the area within 10 cm from the walls.

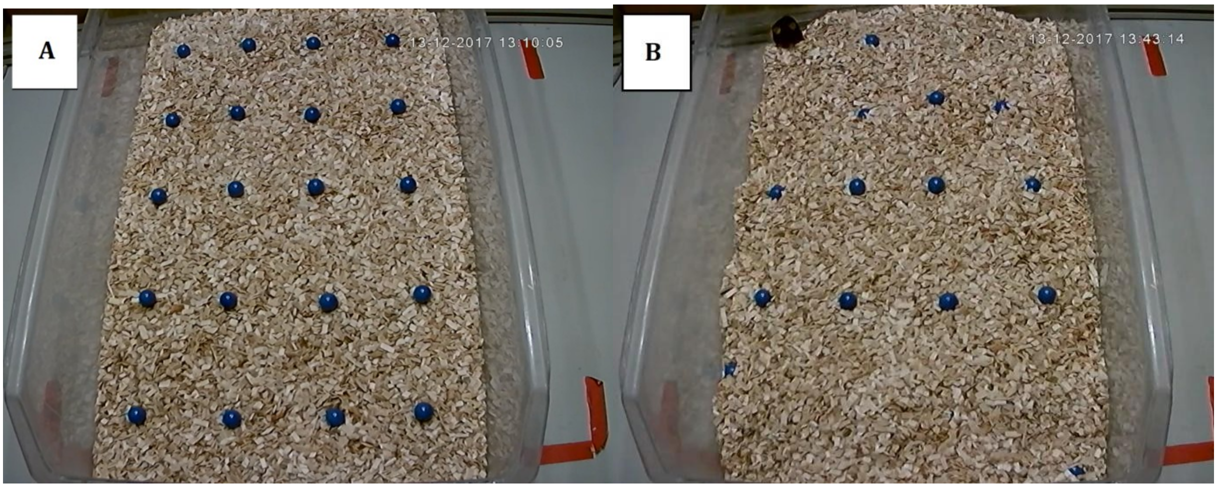

Fig S10. Marble burying behavioral test. A polycarbonate rat cage (26x48x20cm) with mouse bedding material (depth 5 cm) is used. Twenty glass toy marbles are placed in 5 rows of 4 marbles on the bedding surface. During the experiment the top of the cage is covered with a transparent plexiglass® lid, to avoid the escape of the animal. Pictures (a) and (b) were captured by the video camera used for the experiment recording**. (a)** features the set-up before the experiment and (**b)** shows the end phase of the experiment (for both pictures lid was removed for better quality).

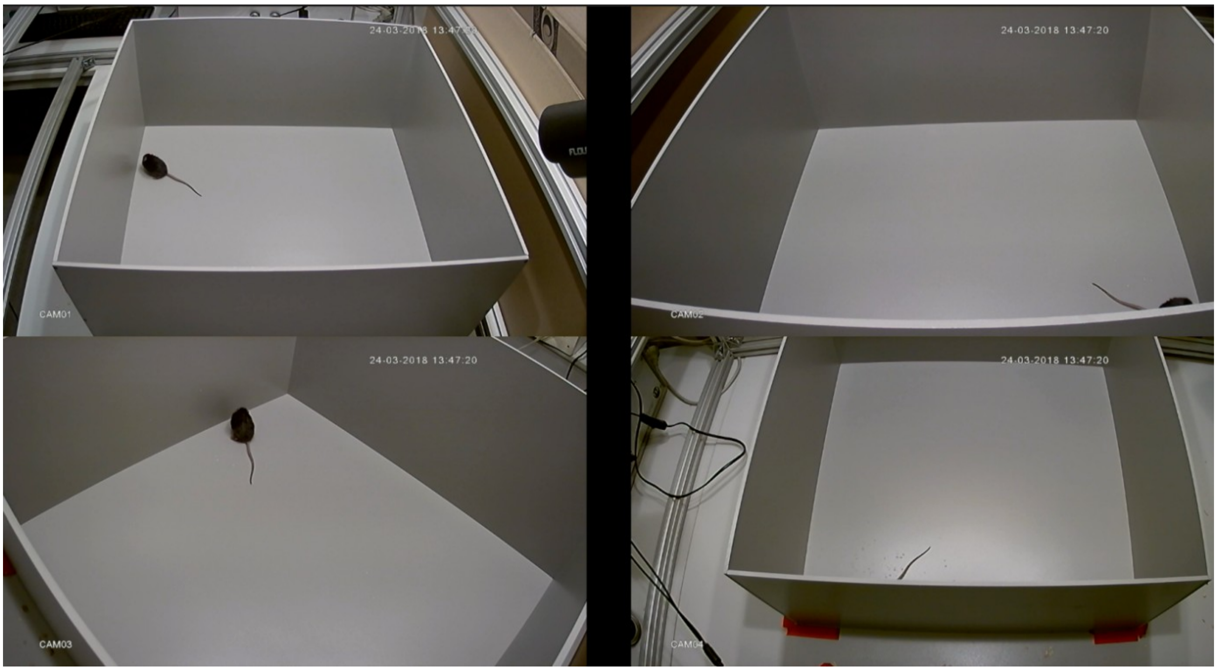

Fig S11. Four side view on the OF activity arena used for the grooming analysis The four videos are used simultaneously to track the animal behavior during the grooming assessment. This design allows a side view on the animal and, ultimately, tackles the grooming microstructure. For the video rendering, freely available software packages (Awesome Video Player © and VLC media player) were used.

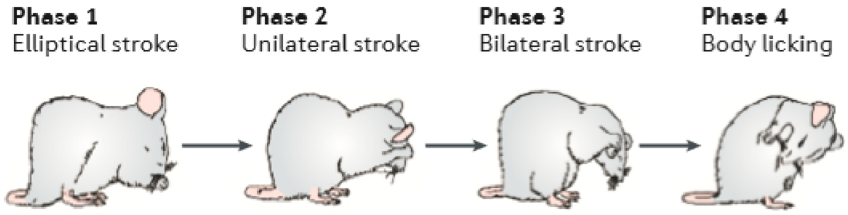

Fig S12. Grooming microstructure adapted from[1] **.** Four phases of the grooming pattern are shown. For the analysis, the extension of these phases proposed in [2] was used: (0) no grooming (not shown), (1) paw licking (phase 1), (2) nose/face/head wash (phases 2-3)(3) body grooming (phase 4), (4) leg licking (not shown), (5) tail/genitals grooming (not shown)

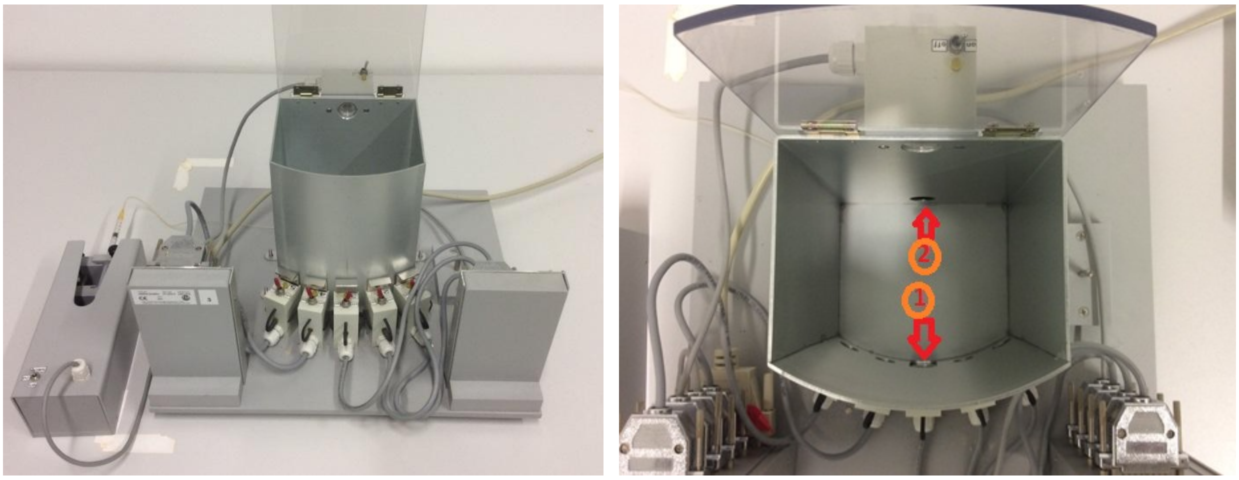

Fig S13. "5 Hole Nose Poke" apparatus

(a) The side photo of 5HNP TSE system. The water is supplied via the mechanical dispenser with the inserted syringe (on the left side) and the experimental workflow is controlled via the computer-assiseted

interface (not shown). **(b)** “1” points to the Hole №3 and “2” refers to the Hole №6. Each hole is equipped with infrared sensors to detect the nose pokes and a light diode. The other holes are closed in this set-up. Mice were put in the box and the training schedule (see Table S2) was automatically executed via computer-based interface.

Tables

Table S1. Statistical analysis of the social interaction. The table shows the mean values of the social interaction parameters time spent for the social interaction and the number of visits in the social interaction zone for all experimental groups: Control, *T. gondii* and *T. gondii*+ Sulfa Post-Infection with the comparison between novel and familiar strangers.

Experimental groups	parameter	Mean value±SEM	Test statistics (paired Student t-test or Wilcoxon signed rank test)	P-value (paired Student t-test or Wilcoxon signed rank test)
Control (n=26)	Time with novel (s)	27±2	t(25)=3.46	0.002
	Time with familiar (s)	17±1.5		
	Visits to novel (n)	71±7.5	t(25)=3.1	0.005
	Visits to familiar (n)	41±5		
T. gondii (n=22)	Time with novel (s)	30±5	t(21)=0.38	0.7
	Time with familiar (s)	27±4		
	Visits to novel (n)	81±23	Z=-0.11	0.9
	Visits to familiar (n)	64±10		
T. gondii+ Sulfa (n=17)	Time with novel (s)	35±6	t(16)=1.97	0.07
	Time with familiar (s)	21±3		
	Visits to novel (n)	59±8.5	t(16)=-0.04	0.97
	Visits to familiar (n)	59±11		

Table S2. Statistical analysis for the total distance traveled. Table represents the total distance traveled in the OF test±SEM (m) and the results of the statistical analysis.

Experimental group	Average distance±SEM (m)	ANOVA (Welch correction if necessary) or	Groups for multiple comparisons	Results of the post-hoc tests:

		Kruskal-Wallis test results		Tukey or Games-Howell for ANOVA .Nemenyui for Kruskal -Wallis
WT (n=25)	60±5	$\chi^2(2)$=10.31, p=0.006	WT vs Toxo	q=3.76, p=0.02
Toxo (n=20)	39±6		WT vs Sulfa	q=0.6, p=0.9
Sulfa (n=16)	63±6		Toxo vs Sulfa	q=4.07, p=0.01

Table S3 Statistical analysis of the exploration strategy. The table compares the time spent in the central compartment±SEM (%) during the first (0-5 min) and the last (10-15 min) 5 min time intervals. For WT and Sulfa the statistical analysis reveals highly significant differences as observed in general, whereas the Toxo group does not show a shift in exploratory behavior.

Experimental group	Time interval	time spent in the central ±SEM (%)	Results of paired Student t-test or Wilcoxon signed rank test.
WT (n=23)	0-5 min	9±1	t(22)= - 6.27, p<0.0001
	10-15 min	20±2	
Toxo (n=20)	0-5 min	15±3	t(19)=0.16.5, p=0.87
	10-15 min	14±4	
Sulfa (n=17)	0-5 min	10±1	t(16)= - 6.44, p<0.0001
	10-15 min	21±2	

Table S4 Statistical analysis of the exploration strategy. Table shows the average time spent in the central compartment±SEM (%) during 5 min intervals in the OF test and the results of the statistical analysis.

Time interval	Experimental group	Time spent in the central compartment ±SEM (%)	ANOVA (Welch correction if necessary) or Kruskal-Wallis test results	Groups for multiple comparisons	Results of the post-hoc tests: Tukey or Games-Howell for ANOVA .Nemenyui for Kruskal -Wallis
0-5 min	WT (n=23)	9±1	$\chi^2(2)$=1.9, p=0.41	WT vs Toxo	-
	Toxo (n=20)	15±3		WT vs Sulfa	-
	Sulfa (n=17)	10±1		Toxo vs Sulfa	-
5-10 min	WT (n=23)	19±2	$\chi^2(2)$=16.9, p=0.0002	WT vs Toxo	Q=4.96,p=0.001
	Toxo (n=20)	10±3.5		WT vs Sulfa	Q=0.51,p=0.93

	Sulfa (n=17)	19±2		Toxo vs Sulfa	Q=5.09,p=0.001
10-15 min	WT (n=23)	20±2	$\chi^2(2)=12.34$, p=0.002	WT vs Toxo	Q=3.89,p=0.02
	Toxo (n=20)	14±4		WT vs Sulfa	Q=1.05,p=0.74
	Sulfa (n=17)	21±2		Toxo vs Sulfa	Q=4.62,p=0.003

Table S5. Statistical analysis for the area preference. The time spent in each area is shown. If the omnibus test (column №4) revealed a statistically significant result, multiple comparisons (column №5) were performed.

Compartment	Experimental group	Time±SEM (% of total time)	ANOVA (Welch correction if necessary) or Kruskal-Wallis test results	Groups for multiple comparisons	Results of the post-hoc tests: Tukey or Games-Howell for ANOVA .Nemenyui for Kruskal -Wallis
Corners	WT (n=23)	43±1.5	$\chi^2(2)=1.84$ p=0.4	WT vs Toxo	-
	Toxo (n=20)	46±5		WT vs Sulfa	-
	Sulfa (n=17)	41±2		Toxo vs Sulfa	-
Center	WT (n=23)	16±1	$\chi^2(2)=6.57$ p=0.04	WT vs Toxo	q=2.85, p=0.11
	Toxo (n=20)	13±3		WT vs Sulfa	q=0.74,p=0.86
	Sulfa (n=17)	17±1.5		Toxo vs Sulfa	q=3.36,p=0.05
Out middle	WT (n=23)	41±1	F(2,57)=0.03, p=0.97	WT vs Toxo	-
	Toxo (n=20)	41±4		WT vs Sulfa	-
	Sulfa (n=17)	42±1		Toxo vs Sulfa	-

Table S6. Digging activity. Table represents the mean values±SEM (column №3) of the digging parameters (column №1) for all experimental groups (column №2). If the omnibus test (column №4) revealed a statistically significant result, multiple comparisons (column №5) were performed.

Experimental parameter	Experimental group	Average distance value±SEM	ANOVA (Welch correction if necessary) or Kruskal-Wallis test results	Groups for multiple comparisons	Results of the post-hoc tests: Tukey or Games-Howell for ANOVA .Nemenyui for Kruskal – Wallis
	WT (n=26)	64±4	$\chi^2(2)=46.7$,	WT vs Toxo	q=9.7, p<0.0001

Number of digging bouts	Toxo (n=27)	1±0.3	p<0.0001	WT vs Sulfa	q=3.7,p=0.03
	Sulfa (n=22)	36±5		Toxo vs Sulfa	q=5.1, p=0.001
Average bout duration (s)	WT (n=26)	4±0.3	$\chi^2(2)=28.1$, p<0.0001	WT vs Toxo	q=6.6,p<0.0001
	Toxo (n=27)	1.2±0.3		WT vs Sulfa	q=0.4, p=0.96
	Sulfa (n=22)	4.5±0.8		Toxo vs Sulfa	q=6.3, p<0.0001
Latency to onset (s)	WT (n=26)	190±20	$\chi^2(2)=22.15$, p<0.0001	WT vs Toxo	q=6.7, p<0.0001
	Toxo (n=27)	850±100		WT vs Sulfa	q=2.4, p=0.21
	Sulfa (n=22)	300±60		Toxo vs Sulfa	q=4.1,p=0.01
Total digging time (s)	WT (n=26)	260±30	$\chi^2(2)=43.2$, p<0.0001	WT vs Toxo	q=9.2, p<0.0001
	Toxo (n=27)	2.5±1		WT vs Sulfa	q=2.6, p=0.17
	Sulfa (n=22)	160±30		Toxo vs Sulfa	q=5.8, p=0.0002

Table S7. Grooming activity. Table represents the mean values±SEM (column №3) of the grooming parameters (column №1) for all experimental groups (column №2). If the omnibus test (column №4) revealed a statistically significant result, multiple comparisons (column №5) were performed.

Experimental parameter	Experimental group	Average distance value±SEM	ANOVA (Welch correction if necessary) or Kruskal-Wallis test results	Groups for multiple comparisons	Results of the post-hoc tests: Tukey or Games-Howell for ANOVA .Nemenyui for Kruskal – Wallis
Number of grooming bouts	WT (n=26)	21±1	F(2,60)=1.2, p=0.3	WT vs Toxo	-
	Toxo (n=20)	18±1		WT vs Sulfa	-
	Sulfa (n=17)	20±2		Toxo vs Sulfa	-
Number of transitions	WT (n=26)	92±3.5	F(2,60)=1.7, p=0.19	WT vs Toxo	-
	Toxo (n=20)	81±6		WT vs Sulfa	-
	Sulfa (n=17)	91±5		Toxo vs Sulfa	-
Percentage of correct transitions (%)	WT (n=26)	42±1	F(2,60)=6.9, p=0.002	WT vs Toxo	Q=5.0,p=0.002
	Toxo (n=20)	34±2		WT vs Sulfa	Q=0.7,p=0.89
	Sulfa (n=17)	41±2		Toxo vs Sulfa	Q=3.9,p=0.02

Total grooming time (s)	WT (n=26)	170±10	F(2,60)=5.05, p=0.009	WT vs Toxo	Q=3.6,p=0.037
	Toxo (n=20)	220±20		WT vs Sulfa	Q=1,p=0.76
	Sulfa (n=17)	150±15		Toxo vs Sulfa	Q=4.2,p=0.012

Table S8. Training schedules used for behavior flexibility test
Table contains the scheme (column №2) of each training schedule (column №1) used in the behavior flexibility test. Each schedule began with 3 minute habituation time (not included in the maximum duration time) followed by the training schedule. The schedule is terminated if the maximum number of rewards (column №3) was consumed or time allocated to the schedule (column №4) expired.

	Order	Maximum number of rewards	Maximum duration
Random Time (RT)	Light№6 and Water delivery → Poke№6 → 60 seconds break (varied stochasticly) → repetition	15	25 minutes
Continuous Reinforcement (CRF)	Light№3 → Poke№3 → Light№6 and water delivery → repetition	15	60 minutes
Random Ratio N	Light№3 → Poke№3 N times (average) → Light№6 and water delivery → repetition	8	60 minutes
Random Interval M	Light№3 → Poke№3 → M seconds (average) → Light№6 and water delivery→ repetition	8	60 minutes
Devaluation test	Light№3 is constantly on	Rewards are not delivered	10 minutes

Table S9
Table contains the results of the behavioral flexibility test comparison between the groups corresponding to the Figure 2.

Experimental group	Experimental schedule	Average normalized poke rate±SEM	Repeated measures ANOVA results	Displayed results for post-hoc comparisons:	Results of the Tukey HDS post-hoc comparison
WT (n=13)	RI-valued	0.55±0.05	F(3,36)=3.85, p=0.017	RI-valued vs RI-devalued	Q=1.56, p=0.69
	RI-devalued	0.45±0.05			
	RR-valued	0.63±0.05			

	RR-devalued	0.37±0.05		RR-valued vs RR-devalued	Q=4.54, p=0.014
Toxo (n=6)	RI-valued	0.79±0.1	F(3,15)=7.15, p=0.003	RI-valued vs RI-devalued	Q=5.43, p=0.008
	RI-devalued	0.21±0.1			
	RR-valued	0.7±0.1		RR-valued vs RR-devalued	Q=3.65, p=0.09
	RR-devalued	0.3±0.1			
Toxo+Sulfa (n=13)	RI-valued	0.59±0.07	F(3,36)=4.82, p=0.006	RI-valued vs RI-devalued	Q=2.6, p=0.27
	RI-devalued	0.41±0.07			
	RR-valued	0.67±0.05		RR-valued vs RR-devalued	Q=4.7, p=0.01
	RR-devalued	0.33±0.05			

Table S10. Changes in the gene expression in Toxo and Sulfa group compared to WT. Table contains the results of the gene expression analysis from *T. gondii* and *T. gondii*+Sulfa experimental group in comparison to Control. Shades of red color correspond to the down-regulation, whereas olive hues depict the degree of up-regulation. Brightness reflects the degree of up or down regulation with bright colors corresponding to the higher degree of alternation of gene expression. To minimize potential experimental biases, we suggest relying on the data patterns reproduced in both cohorts (i.e. Cohort 1 and Cohort 2).

Full name	Symbol	Toxo		Sulfa	
		Cohort №2	Cohort №1	Cohort №2	Cohort №1
Beta-2-microglobulin	B2m	44.25	42.91	51.6	17.94
Prostogladin-endoperoxide synthase 2	Ptgs2	21.9	4.35	6.78	2.03
Glial fibrillary acidic protein	Gfap	11.69	11.03	12.5	6.77
Phosphoinositide-3-kinase, catalytic, gamma polypeptide	Pik3cg	8.13	5.36	6.44	5.25
Tryptophan hydroxylase 1	Tph1	3.42	2.58	0.47	0.7
Phospholipase C, beta 2	Plcb2	3.39	2.5	3.46	2.25
Phospholipase A2, group V	Pla2g5	2.84	2.59	1.62	2.24
Glucuronidase, beta	Gusb	2.5	4.22	1.89	2.8
Interleukin 1 beta	Il1b	258.15	190.55	109.32	16
Adenylate cyclase 7	Adcy7	8.06	3.57	5.08	4
Ceroid lipofuscinosis, neuronal 3	Cln3	5.08	6.70	2.67	0.5
Arginine vasopressin	Avp	4.19	51.41	4.27	0.56
Purinergic receptor P2X, ligand-gated ion channel, 7	P2rx7	3.40	9.95	4.35	4
Glutamate receptor, metabotropic 6	Grm6	3.27	20.72	4.15	0.08
Adenosine A1 receptor	Adora1	2.86	2.52	2.05	1
Brain derived neurotrophic factor	Bdnf	2.25	3.55	1.68	0.15
Solute carrier family 7 (cationic amino acid transporter), member 11	Slc7a11	2.22	15.86	3.11	4
Solute carrier family 1 (glial high affinity glutamate transporter), member 3	Slc1a3	2.00	9.70	1.6	1
Phosphodiesterase 10A	Pde10a	0.22	0.3	0.13	0.75
Dopamine receptor D1A	Drd1a	0.27	0.25	0.17	0.73
5-hydroxytryptamine (serotonin) receptor 1D	Htr1d	0.3	0.35	0.26	0.89
5-hydroxytryptamine (serotonin) receptor 6	Htr6	0.31	0.26	0.3	0.8
Dopamine receptor D5	Drd5	0.35	0.25	0.84	0.64
Protein phosphatase 1, regulatory (inhibitor) subunit 1B	Ppp1r1b	0.35	0.25	0.21	0.89
5-hydroxytryptamine (serotonin) receptor 1B	Htr1b	0.37	0.51	0.3	0.73
Phospholipase C, beta 1	Plcb1	0.37	0.41	0.29	0.76
Inositol 1,4,5-trisphosphate receptor 1	Itpr1	0.47	0.31	0.28	0.78
Dopamine receptor D2	Drd2	0.47	0.28	0.29	1
Synuclein, alpha	Snca	0.48	0.36	0.49	0.72
Adenylate cyclase 5	Adcy5	0.51	0.12	0.47	0.65
Calcium channel, voltage-dependent, P/Q type, alpha 1A subunit	Cacna1a	0.53	0.25	0.55	0.79
Solute carrier family 6 (neurotransmitter transporter, dopamine), member 3	Slc32a1	0.49	0.40	0.88	0.5

Table S11. Statistical tests applied. Table provides the analysis strategy for all data types, regarding normality/multivariate normality and homoscedasticity/sphericity, and various types of data sets (paired groups, unpaired groups, multiple groups)

Normality/homoscedasticity		Yes	No
Yes	Two unpaired groups	Unpaired Student's t-test[3]	Welch's t-test for two unpaired groups[4]

	Two paired groups		Paired Student's t-test[3]	
	Multiple groups (n ≥3)	Unpaired groups	One-way ANOVA, with Tukey post hoc tests[5]	Welh's ANOVA test[6] with Games-Howel post hoc test [7]
		Paired groups	Repeated measures ANOVA with Tukey post hoc test	Repeated measures ANOVA with corrected degrees of freedom or MANOVA
No	Two unpaired groups		Mann-Whitney U-test[8,9]	
	Two paired groups		Wilcoxon Signed Rank Test[10]	
	Multiple groups (n ≥3)	Unpaired groups	Kruskal-Wallis rank-sum test[11] with Nemenyui as a post hoc test	
		Paired groups	Friedman's ANOVA test [12]with Wilcoxon Signed Rank Test for pairwise comparison with multiple comparison correction (e.g. Holm-Bonferroni correction[13])	

Supplementary References

[1] Kalueff A V, Stewart AM, Song C, Berridge KC, Graybiel AM, Fentress JC. Neurobiology of rodent self-grooming and its value for translational neuroscience. Nat Rev Neurosci 2016;17:45–59. https://doi.org/10.1038/nrn.2015.8.

[2] Kalueff A V, Aldridge JW, LaPorte JL, Murphy DL, Tuohimaa P. Analyzing grooming microstructure in neurobehavioral experiments. Nat Protoc 2007;2:2538–44. https://doi.org/10.1038/nprot.2007.367.

[3] Student. The Probable Error of a Mean. Biometrika 1908;6:1–25. https://doi.org/10.2307/2331554.

[4] Welch BL. The Generalization of `Student's' Problem when Several Different Population Variances are Involved. Biometrika 1947;34:28–35. https://doi.org/10.2307/2332510.

[5] Tukey JW. Comparing Individual Means in the Analysis of Variance. Biometrics 1949;5:99–114. https://doi.org/10.2307/3001913.

[6] WELCH BL. ON THE COMPARISON OF SEVERAL MEAN VALUES: AN ALTERNATIVE APPROACH. Biometrika 1951;38:330–6. https://doi.org/10.1093/biomet/38.3-4.330.

[7] Games PA, Howell JF. Pairwise Multiple Comparison Procedures with Unequal N's and/or Variances: A Monte Carlo Study. Journal of Educational Statistics 1976;1:113–25. https://doi.org/10.2307/1164979.

[8] Mann HB, Whitney DR. On a Test of Whether one of Two Random Variables is Stochastically Larger than the Other. The Annals of Mathematical Statistics 1947;18:50–60.

[9] Fay MP, Proschan MA. Wilcoxon-Mann-Whitney or t-test? On assumptions for hypothesis tests and multiple interpretations of decision rules. Stat Surv 2010;4:1–39. https://doi.org/10.1214/09-SS051.

[10] Wilcoxon F. Individual Comparisons by Ranking Methods. Biometrics Bulletin 1945;1:80–3. https://doi.org/10.2307/3001968.

[11] Kruskal WH, Wallis WA. Use of Ranks in One-Criterion Variance Analysis. J Am Stat Assoc 1952;47:583–621. https://doi.org/10.2307/2280779.

[12] Friedman M. The Use of Ranks to Avoid the Assumption of Normality Implicit in the Analysis of Variance. J Am Stat Assoc 1937;32:675–701. https://doi.org/10.1080/01621459.1937.10503522.

[13] Holm S. A Simple Sequentially Rejective Multiple Test Procedure. Scandinavian Journal of Statistics 1979;6:65–70.
